# Restorative perceptions of migratory elderly: insights for inclusive urban park design in china

**DOI:** 10.3389/fpsyg.2025.1695092

**Published:** 2025-11-28

**Authors:** Yurui Chen, Shureen Faris Abdul Shukor, Suhardi Maulan, Adam Aruldewan S. Muthuveeran

**Affiliations:** 1 Department of Urban and Rural Planning and Landscape Architecture, School of Architecture and Civil Engineering, Xihua University, Chengdu, China; 2 Department of Landscape Architecture, Faculty of Design and Architecture, Universiti Putra Malaysia, Serdang, Selangor, Malaysia

**Keywords:** elderly migration, restorative environments, attachment, design strategies, urban parks

## Abstract

**Introduction:**

Migratory elderly—older adults who relocate to urban areas to live with their children—constitute a growing demographic shaped by global aging and rapid urbanization. Yet their restorative experiences in everyday urban nature remain under-examined. This study investigates their preferred spatial features, factors shaping restorative perceptions, and design recommendations to enhance restorative outcomes in urban parks.

**Methods:**

We conducted semi-structured interviews with 23 migratory elderly in two parks in Chengdu, China, and applied thematic analysis.

**Results:**

Restorative perceptions clustered around three themes: (1) personal needs; (2) place-related memory and identity; and (3) preferred spatial features. Compared with non-migrants, the migratory elderly exhibited a stronger reliance on culturally familiar cues—such as native vegetation, agrarian water features, and traditional forms—which evoked nostalgia and supported attachments to both past and present places. Although participants employed self-regulatory strategies and formed new attachments, unmet needs and usability conflicts (e.g., noise, activity clashes, and unclear wayfinding) constrained restorative benefits.

**Discussion and conclusions:**

Theoretically, extensions to restorative-environment frameworks include the introduction of migration-related discontinuities as boundary conditions, the identification of a practice- and community-mediated pathway to accelerated attachment, and the specification of a chain linking inclusive design to preferences, then to attachment, and ultimately to perceived restorativeness. Practically, we recommend a five-stage approach: (1) Planning—participatory framing to balance active and quiet zones; (2) Spatial structure—a legible hierarchy with intuitive, barrier-free links; (3) Facilities—provision of shade, seating, toilets, and lighting to balance recreation with safety and accessibility; (4) Social management—programming for solitary and group use that fosters cultural resonance; and (5) Maintenance—feedback-driven adaptation with clear wayfinding and night-time illumination. These findings underscore the value of culturally sensitive, inclusive park design in supporting psychological restoration among the migratory elderly in rapidly urbanizing societies.

## 1 Introduction

Population aging is an accelerating global trend, with an estimated 1.5 billion people aged 65 and above by 2050 (Navaneetham and Arunachalam, 2022). China, home to a rapidly aging population, is also experiencing a particularly rapid demographic shift. With ongoing urbanization, older adults who relocate to other cities have increasingly attracted scholarly and policy attention (Holecki et al., 2020). Many move from their hometowns to urban areas primarily to reunite with family or care for grandchildren (Wu and Wu, 2023; Fang et al., 2015). This internal migration, referred to as migratory elderly (Hu et al., 2023; MacLachlan and Gong, 2022), entails distinct adaptation demands compared to non-migratory peers. Relocation can disrupt life continuity, limit access to familiar resources, and heighten risks of isolation, loneliness, and anxiety (Li et al., 2017). Migratory elderly also face barriers in accessing urban services, local health-care utilization (Zhang et al., 2018), alongside challenges in adapting to new norms and rebuilding social networks (Tang et al., 2020).

The way migratory elderly cope with these challenges is closely tied to their perception of the living environment. A strong sense of urban identity has been shown to improve health outcomes among migratory elderly (Chi, 2021), whereas negative evaluations of the environment are linked to poorer self-rated health (Jin et al., 2020). Identity formation in the new urban context is therefore integral to the well-being of migratory elderly (Jagroep et al., 2023). Against this backdrop, urban parks represent vital public resources. They are widely recognized as restorative environments that alleviate stress, support social interaction, and enhance psychological health. “Restorative” in this sense refers to the capacity of natural and designed environments to facilitate recovery from mental fatigue and stress (Ulrich et al., 1991; Kaplan and Kaplan, 1989). Empirical studies have demonstrated that viewing or visiting urban parks fosters physiological relaxation and psychological restoration (Pratiwi et al., 2019), while reinforcing place attachment (Liu et al., 2021; Gidlöf-Gunnarsson and Öhrström, 2007).

However, the migration patterns of elderly populations in China differ substantially from those in Western countries. Whereas older adults in many Western contexts relocate from urban to rural areas for retirement (Bloem et al., 2008), Chinese elderly commonly migrate from rural to urban settings, particularly to metropolitan centers and frontier provinces (Dou and Liu, 2017). This pattern is shaped by intergenerational family structures influenced by the one-child policy and disparities in pension systems (Wu and Wu, 2023). According to the PRC Health and Family Planning Commission (2014), internal migration of elderly individuals has become an important social phenomenon with profound implications for urban planning and public health. Comparable challenges have been documented internationally: migratory elderly in Germany exhibit higher risks of malnutrition (Paker-Eichelkraut et al., 2013), while in India, migratory elderly report worse health outcomes compared to their non-migrant counterparts (Mandal and Pradhan, 2024).

Within China, middle-aged and older adults constitute the majority of urban park users (Cohen et al., 2019; Binbin et al., 2012). Their preferences differ markedly from those of younger populations, with specific park features strongly shaping frequency of visits, levels of physical activity, and opportunities for social engagement (Zhang et al., 2022; Onose et al., 2020; Veitch et al., 2020). Parks designed with older adults in mind can better mitigate risks of social isolation and enhance wellbeing (Loukaitou-Sideris et al., 2016). Yet, despite extensive research on the restorative benefits of urban parks and the leisure preferences of elderly populations, most existing studies have focused on local elderly or the general urban public, largely overlooking the experiences of migratory elderly—a group defined by both aging and migration.

Given their dual identity, migratory elderly may perceive and use parks in ways that diverge from long-term residents. Their restorative experiences may be deeply shaped by memory, mobility, and cultural traditions, yet empirical evidence on these processes remains scarce, especially in the Chinese context. While previous studies have recognized the restorative potential of parks, few have explicitly examined how migration reshapes place attachment and spatial preferences in later life. To address this gap, this study develops an integrated perspective linking mobility, memory, and cultural identity to the restorative functions of urban parks. Specifically, it investigates the restorative perception mechanisms of the migratory elderly in Chengdu, China, and proposes design strategies tailored to their cultural backgrounds and emotional needs. The research questions were as follows: (1) What are the preferred spatial features for the migratory elderly? (2) What are the factors that support the migratory elderly’s restorative perception in urban parks? (3) What are the urban park design recommendations for the migratory elderly?

## 2 Literature review

### 2.1 Inclusive urban park design for aging and mobility contexts

Inclusive urban park design represents a crucial dimension of wellbeing for older adults. Liu et al. (2020) demonstrated that well-designed park amenities significantly enhance opportunities for social interaction, thereby contributing to physical and psychological benefits. Similarly, in depopulated urban communities, residents emphasize the value of small, enclosed green spaces for fostering stronger social connections (Francisca Lima et al., 2020). Inclusive design emphasizes accessibility, safety, and social participation, ensuring that diverse user groups—including migratory elderly—can benefit equitably from public green spaces (Ordóñez-Barona, 2017). For migratory elderly in China, parks serve not only as restorative environments but also as social and cultural platforms that facilitate adaptation and identity reconstruction. These findings align with broader evidence that social support, subjective wellbeing, and resilience promote positive social adjustment among seniors (Yu et al., 2024).

Existing studies predominantly examine general older populations, often assuming residential continuity and stable social networks. Mobility-related discontinuities—such as disrupted routines, unfamiliar norms, and weak local ties—have received limited attention. Consequently, little is known about how inclusive design features translate into perceived restorativeness when familiarity and social anchoring are fluid for migratory elderly. Moreover, demographic and social background factors (e.g., age, education, income) may influence park use, further shaping restorative experiences.

### 2.2 Landscape preferences and place attachment among migratory elderly

In terms of spatial preferences, seniors tend to favor pathways, paved open spaces, and natural areas (Zhai et al., 2021). However, evidence specific to migratory elderly remains limited, with indications that their preferences diverge from residents (Dai et al., 2023; Kaixuan and Min, 2022; Sun Wenshu, 2021). In the Chinese context, Yang et al. (2020) further highlighted distinctive pathways through which residential green space influences the mental health of migratory elderly. Collectively, these findings underscore the necessity of examining the specific environmental preferences of migratory elderly in order to inform inclusive and responsive park design.

Place attachment describes the emotional and functional ties that individuals develop with specific environments (Scannell and Gifford, 2010b), which refers to the extent to which individuals’ functional and emotional needs are fulfilled within a given environment (Bazrafshan et al., 2021). It is dynamic, evolving through practical utility, emotional resonance, and socio-spatial interaction (Ujang and Zakariya, 2015; Moulay et al., 2018). Attachment shapes environmental preferences and the perceived quality of restorative experiences; for example, local nature landscapes tend to be preferred over non-local ones (Bazrafshan et al., 2023; Menatti et al., 2019). Socio-spatial networks, community ties, and the continuity or familiarity of settings further reinforce its evolution (Ellenbogen and Trivic, 2024).

For migratory elderly, the psychological dimensions of place attachment—comprising affective, cognitive, and behavioral components—are especially salient in establishing a sense of belonging in unfamiliar urban contexts (Palladino, 2019). Western countries show that immigrant experiences in parks can be both inclusive and exclusionary, highlighting how social and cultural contexts shape attachment (Main, 2013). While the length of residence typically strengthens attachment (Lewicka, 2011), this relationship may be weaker or inconsistent among populations with disrupted residential histories, such as the migratory elderly. The attachment may depend more on affordances and inclusive social cues than on time. Collectively, these insights suggest that landscape preference and attachment act jointly—preferences shape initial engagement, whereas attachment consolidates emotional restoration and identity continuity.

### 2.3 Restorative perception and environmental psychology foundations

Restoration theory posits that environmental qualities support attentional recovery and stress reduction (Kaplan, 1995). According to Attention Restoration Theory (ART), four environmental attributes—coherence, complexity, mystery, and legibility—foster restorativeness through cognitive engagement, while being away, fascination, and compatibility describe the perceptual and experiential dimensions (Hartig et al., 1997).

Empirical evidence consistently shows that perceived restorativeness is positively associated with environmental preferences (Abkar et al., 2011; Huang et al., 2008; Kyle et al., 2003). In China, park restorativeness has been linked not only to specific landscape features but also to place attachment, with theoretical models proposed to explain these interrelationships (Liu, 2020). From a psychological perspective, environmental perception, attachment, and restorative experience jointly support self-regulation by providing external resources, stimulating intrinsic motivation, and strengthening coping capacity (Bagozzi, 1992). Despite increasing evidence, few studies have investigated how migration-related unfamiliarity or disrupted social ties moderate these restorative pathways. Understanding this interaction is essential to identifying whether physical improvements alone can compensate for weaker socio-spatial anchoring.

### 2.4 Conceptual framework

**FIGURE 1:**
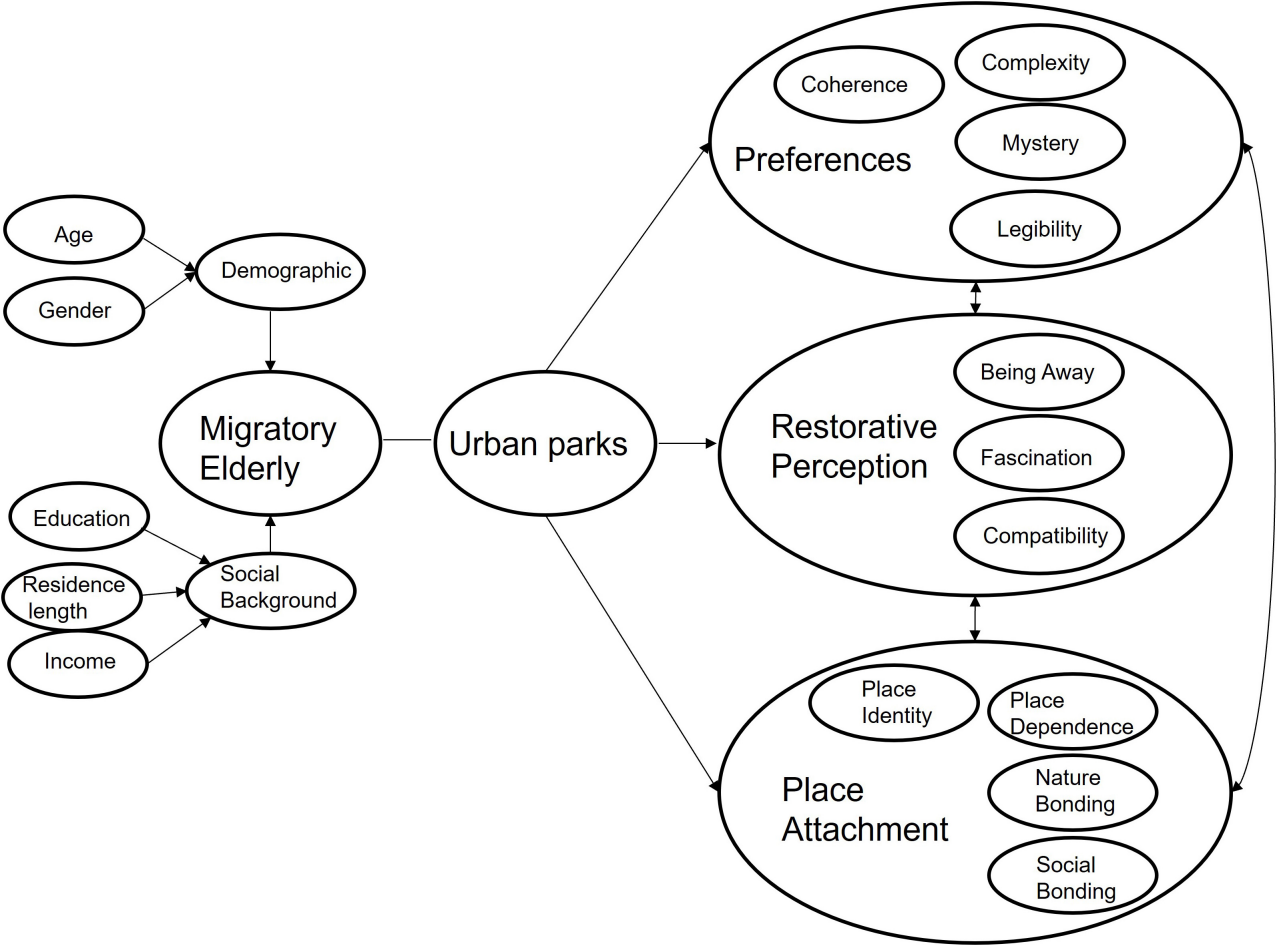
Conceptual framework.

Drawing upon validated quantitative constructs and qualitative insights, this study integrates landscape preference, place attachment, and restorative perception into a unified analytical model (Figure 1). The framework proposes a sequential relationship among these variables within the context of urban park experiences of migratory elderly. Demographic and social background factors (such as age, gender, education, income, and residence length) influence park use, which in turn shapes environmental preferences. Preferences for coherent, legible, and moderately complex features enhance restorative perception through greater feelings of being away, fascination, and compatibility. Place attachment promotes restorative experiences, reflected in growing place identity, place dependence, nature bonding, and social bonding. Attachment subsequently reinforces restorative perception by fostering familiarity, belonging, and emotional security in new urban environments.

Informed by classical restorative environment theories, such as the ART (Kaplan, 1995) and subsequent environmental psychology models (Hartig et al., 1997)—this framework situates the experiences of migratory elderly within conditions of mobility and adaptation. It introduces migration-related discontinuities as boundary conditions that potentially reshape restorative processes, identifies a practice- and community-mediated pathway that enables accelerated place attachment, and specifies a design-anchored sequence linking inclusive urban park features to preferences, attachment, and perceived restorativeness. By integrating these dimensions, the framework advances understanding of how restorative mechanisms operate when familiarity, continuity, and social anchoring are disrupted.

Accordingly, the conceptual framework positions inclusive urban parks as both a physical and social foundation through which preferences and attachments jointly contribute to restorative perception. Figure 1 summarizes these dimensions, and operational definitions are provided in Supplementary material.

## 3 Materials and methods

### 3.1 Design and participants

This study adopted a qualitative research design, employing in-depth, face-to-face interviews to explore the restorative perceptions of migratory elderly in urban parks. The research protocol followed international ethical principles outlined in the Declaration of Helsinki and was approved by the Ethics Committee for Research Involving Human Subjects at Universiti Putra Malaysia in October 2024 (Approval No.: JKEUPM-2024-307).

Before participation, respondents were fully informed of the study’s objectives, procedures, and their right to withdraw at any stage without penalty. Written informed consent was obtained, and participants were assured that audio recordings would be used strictly for academic purposes. To preserve confidentiality, all personal identifiers were removed, and each participant was assigned a unique code (e.g., P1, P2, …, P23) for data management and analysis.

Data collection took place between December 2024 and January 2025 in Chengdu, China. Chengdu was chosen as the study site for three key reasons: (1) Demographic trends: Since 2005, internal migration in China has increasingly included older adults relocating from eastern coastal provinces to central and western regions (Gu et al., 2022). Chengdu has become a significant destination for such flows. (2) Urban context: As China’s first designated “Park City,” Chengdu has incorporated extensive green infrastructure into its urban fabric, offering a highly relevant context for examining park-based wellbeing. The data were mainly sourced from the *Chengdu Municipal Statistics Bureau* (2023). (3) Aging phenomenon: By 2025, 19.1% of Chengdu’s registered population is projected to be aged 65 years or older, rising to 20.56% by 2027, underscoring the urgency of age-friendly urban planning. The data were obtained from the *Report on Chengdu’s Elderly Population Information and Aging Industry Development* (2022).

**FIGURE 2:**
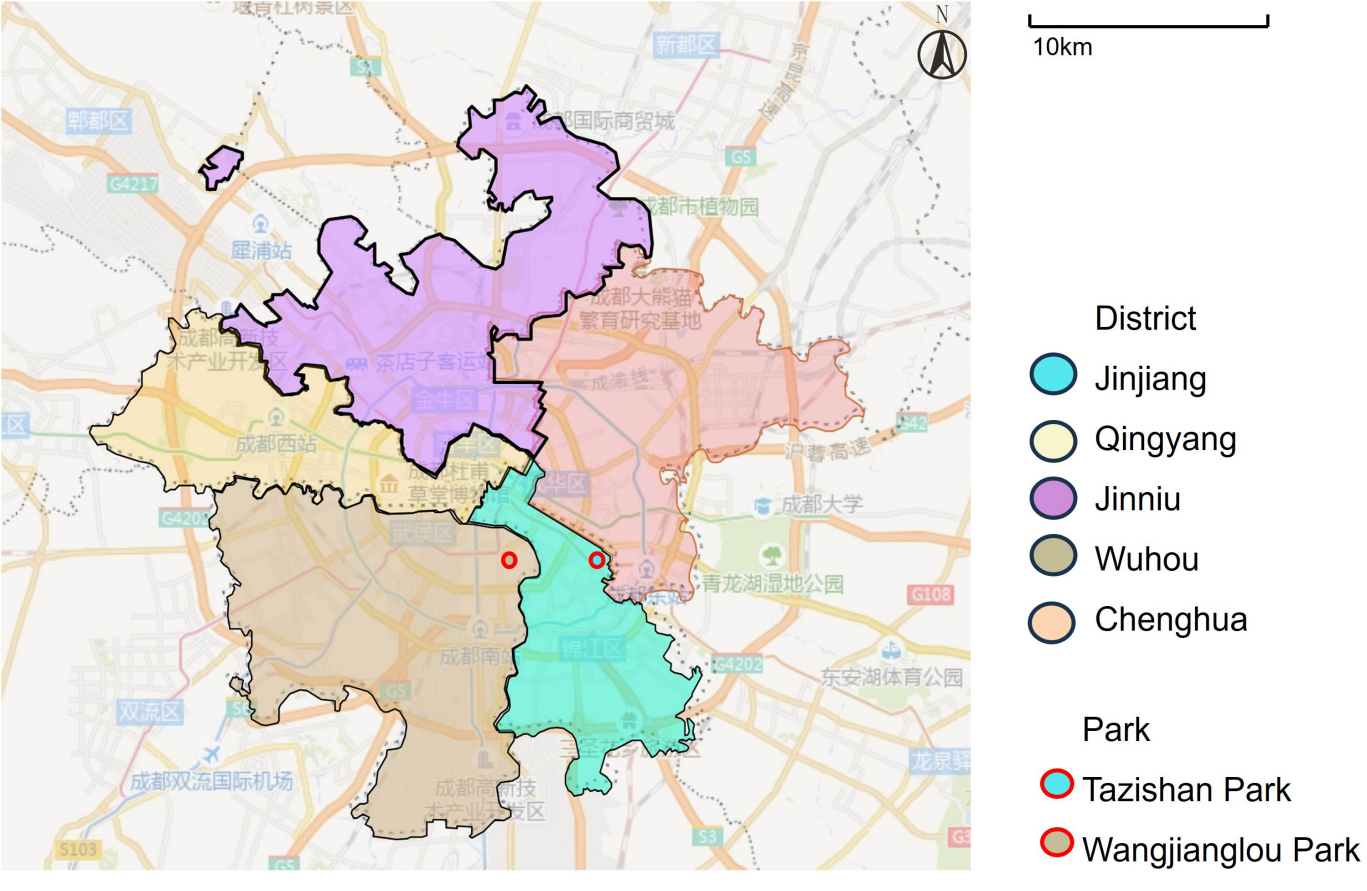
Position of two parks. Map data © OpenStreetMap contributors, ODbL 1.0.

**FIGURE 3:**
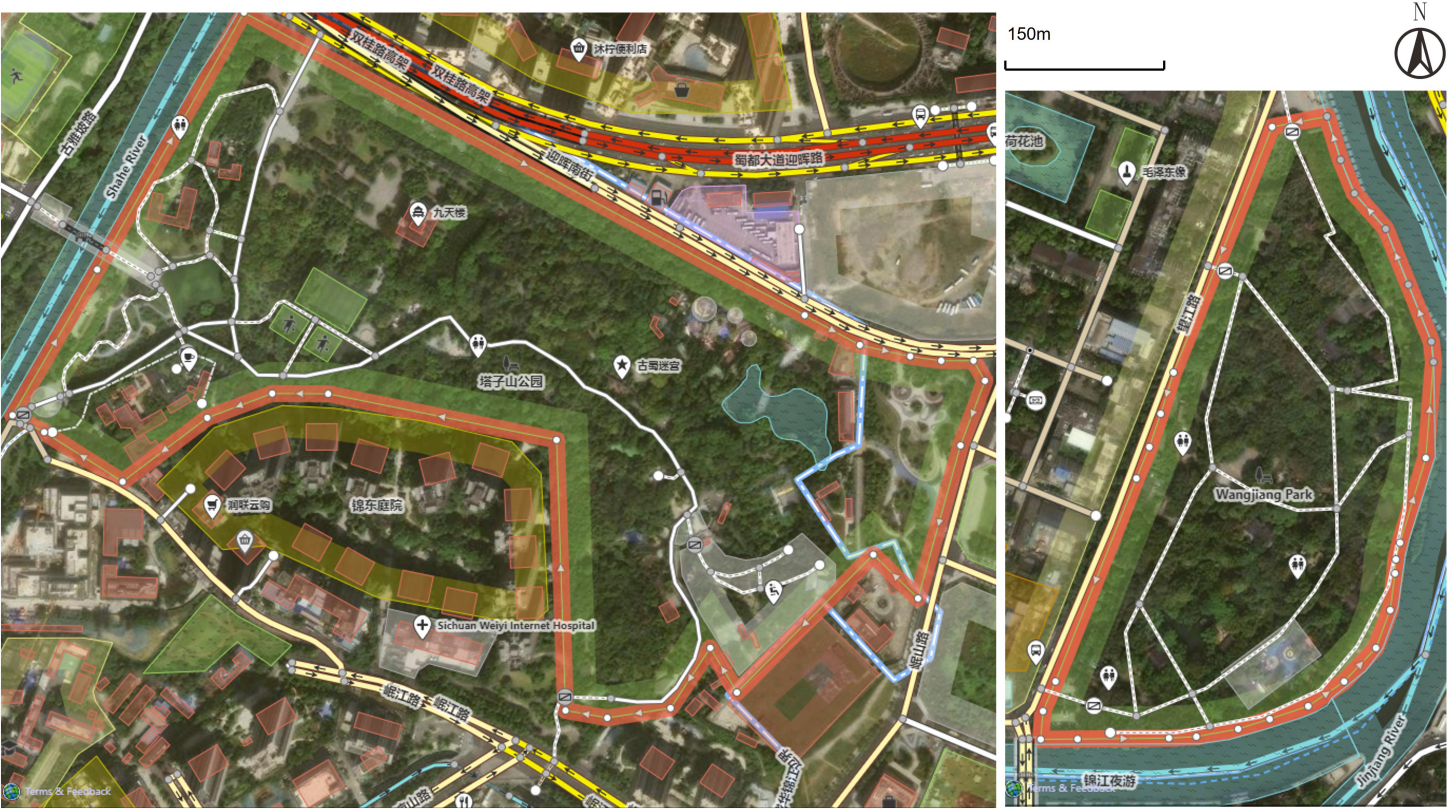
Maps of Tazishan Park (left) and Wangjiang Park (right). Map data © OpenStreetMap contributors, ODbL 1.0.

Two urban parks—Tazi Shan Park (Wuhou District) and Wangjianglou Park (Jinjiang District)—were selected as research sites (see Figure 2). These parks differ in scale, spatial layout, user demographics, and recreational facilities, thus providing contrasting contexts for exploring restorative experiences. Both feature diverse landscapes integrating natural and built elements, such as artificial lakes, rockeries, wetlands, sports courts, and leisure zones. Tazishan Park covers 26.1 hectares and was built in 2011. Wangjianglou Park covers 12.6 hectares and was built in 1889. Map data © OpenStreetMap contributors, ODbL 1.0 (see Figure 3).

The participants were selected using a convenience sampling method. Due to the need to fully immerse themselves in the urban park, the subjects were searched in the urban park. The inclusion criteria were: (1) aged 50 years or above, (2) possessing non-local household registration (hukou) indicating migration from another region, and (3) having visited the park on more than one occasion. This age threshold reflects current trends in China’s migratory elderly, which often includes individuals being relatively young older adults (Wu and Wu, 2023). A total of 23 participants (14 women, 9 men) aged between 50 and 85 years were interviewed.

### 3.2 Instruments

A semi-structured interview guide was developed drawing upon established literature on qualitative interviewing (Wengraf, 2001; Minichiello, 1995), and aligned with the study’s theoretical framework, which focused on environmental perception, landscape preferences, place attachment, and perceived restorativeness. Interviews were initially guided by predefined dimensions and then broadened through follow-up probes to mitigate confirmation bias.

Target constructs and their dimensions were identified *a priori* based on core instruments, including the Environmental Preference Scale (primarily the modified version by Huang et al. (2008), the Place Attachment Scale (Dasgupta et al., 2022; Afshar et al., 2021), and the Restorative Perception Scale (Liu et al., 2018). To ensure theoretical sensitivity while preserving openness, closed-ended scale items were translated into open-ended, contextualized, and behaviorally oriented prompts. For each dimension, we specified a primary question followed by targeted probes and exploratory prompts.

To ensure cultural appropriateness, the guide was reviewed by experts in environmental psychology and urban studies, and subsequently refined through two pilot interviews with migratory elderly not included in the final sample. Adjustments were made to simplify wording, account for dialectal differences, and ensure accessibility for participants with varying educational backgrounds.

All interviews were conducted in Mandarin Chinese within the two selected parks. The recording files are mainly in dialects from Southwest China and other regions (including but not limited to the Sichuan dialect), which are transcribed into Chinese characters. The flexible structure of the guide allowed interviewers to adjust the sequence of questions and introduce follow-up prompts in response to participants’ narratives. With informed consent, all interviews were audio-recorded to ensure accuracy, and supplementary field notes were taken to capture contextual details and non-verbal cues. Each participant’s sessions last approximately 30 min. A brief transcription workflow was applied, involving human transcription followed by a two-pass verification to ensure accuracy and reliability.

### 3.3 Data collection and analysis

In qualitative research, sample size is determined by data saturation rather than statistical power (Smith, 2003; Burmeister and Aitken, 2012). Data saturation was defined as the point at which no new first-order codes emerged across three consecutive interviews and no further revisions to theme boundaries were required (Supplementary material). In this study, recruitment continued until no new themes emerged, which occurred after the 21st interview. Saturation was determined when *N* = 2 consecutive documents had zero new first-level codes and the subject memorandum had not been revised. Thematic saturation was tracked via iterative coding: two researchers independently coded transcripts after each set of five interviews and then compared codes. Saturation was deemed achieved when subsequent interviews produced only repeated codes and overlapping themes across key emotional and behavioral domains. Two additional interviews were conducted to confirm saturation (Saunders et al., 2018), resulting in a final sample of 23 participants.

The research team comprised five members: a female doctoral candidate in urban design (10 years of park design research experience), a female master’s student in landscape design, and three undergraduate volunteers (two male, one female) specializing in landscape architecture. To minimize potential bias, all team members documented their preconceptions and expectations regarding the research topic in reflective journals before data collection. Regular team meetings were held to critically examine the potential influence of researchers’ backgrounds on interpretation. No conflicts of interest were reported.

Semi-structured, in-depth interviews were carried out after participants provided written informed consent. One researcher facilitated the conversation, while another took detailed notes and observed contextual nuances. All interviews were audio-recorded with permission, transcribed verbatim, and subsequently analyzed using MAXQDA 2024 software.

**FIGURE 4:**
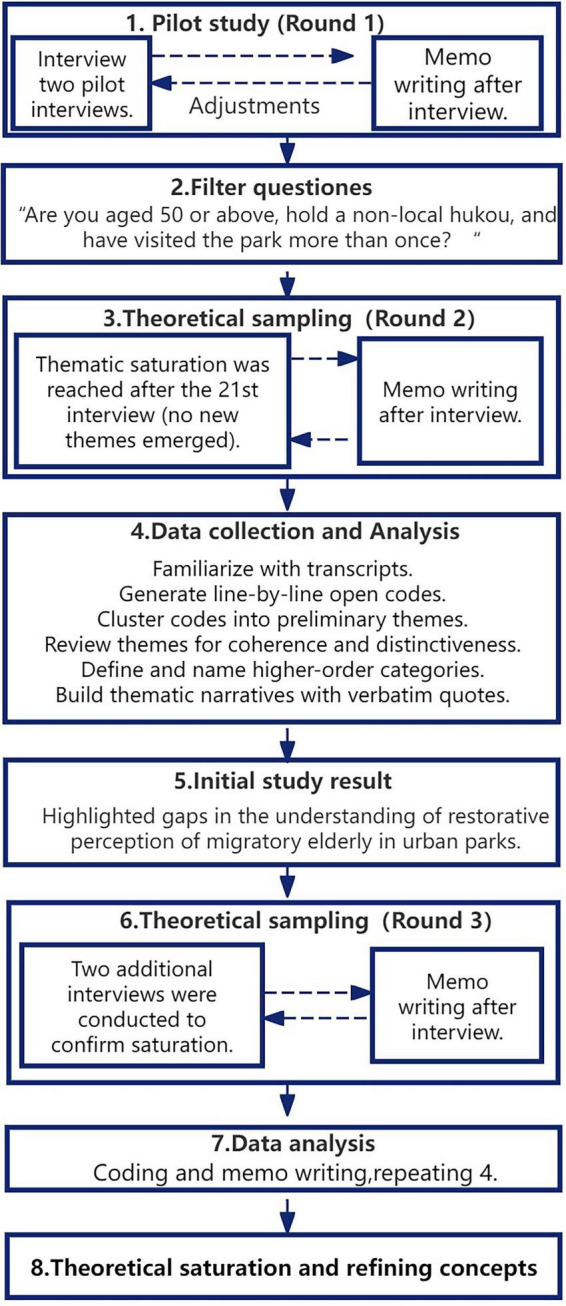
Study design (coding process and saturation determination).

Thematic analysis followed Braun and Clarke’s (2006) six-phase framework, combining inductive (data-driven) and deductive (theory-driven) approaches. The process entailed: (1) familiarization with the transcripts; (2) generating initial open codes through line-by-line reading; (3) clustering related codes into preliminary themes (axial coding); (4) reviewing themes for internal coherence and distinctiveness; (5) defining and naming higher-order categories (selective coding); and (6) constructing thematic narratives supported by verbatim quotations. As shown in step 4 of Figure 4.

To ensure coding reliability, two lead researchers independently coded the transcripts (Charpentier and Kirouac, 2022). Rigor was further enhanced through member checking, whereby participants reviewed transcripts and preliminary interpretations to validate accuracy. Descriptive coding was applied in the first phase of the analysis scheme (S1). Subsequently, broader categories were developed and guided the second and third phases of the analysis (S2 and S3) (Saldaña, 2021). The underlying concepts were drawn from existing theory, but the authors were open to new themes and inter-code relationships. The Kappa value was calculated using the Coder Comparison Queries for coding consistency in this study. The Kappa value for the first round of the coding was 0.73, which indicates substantial consistency. The Kappa value for the second round was 0.86, which is almost perfectly consistent. All authors reviewed the interview transcriptions and participated in thematic discussions with experts from various related fields. Details of the coding process are presented in Supplementary Material 4.

## 4 Results

Guided by the theoretical framework, the analysis identified three interrelated themes concerning restorative cognition among migratory elderly in urban parks: personal needs, place-related memory and identity, and preferred spatial features. These themes illuminate how migratory elderly negotiate urban park spaces to maintain wellbeing and cultural continuity.

### 4.1 Sample characteristics

The final sample comprised 23 participants who demonstrated notable heterogeneity in age, gender, duration of residence in Chengdu, education, income, and cultural background (Table 1). Such diversity provided a robust basis for capturing varied perspectives on park use and restorative experiences. The sample skewed female (60.9%). Women were more represented in the 65–80 cohort (66.7%), while the > 80 group was evenly split.

**TABLE 1:** Respondents’ characteristics and Age cross-tabulation (*N* = 23).

A. Overall characteristics			
Characteristic	Category	Total n	Percentage
Age	50-65	10	43.5
	65–80	9	39.1
	Over 80	4	17.4
Years in Chengdu	1–4	6	26.1
	5–10	8	34.8
	10–15	9	39.1
Gender	Male	9	39.1
	Female	14	60.9
Education	Primary school	2	8.7
	Secondary school	11	47.8
	University diploma	7	30.4
	Postgraduate	3	13.0
Income (RMB/month)	Less than 3000	5	21.7
	3,000–5,000	11	47.8
	Over 5000	7	30.4
**B. Age,× Gender (row% within age group)**			
**Age group**	**Male n (%)**	**Female n (%)**	**Total**
50–65	4 (40)	6 (60)	10
65–80	3 (33.3)	6 (66.7)	9
>80	2 (50)	2 (50)	4
Total	9 (39.1)	14 (60.9)	23

Residency duration varied. This mix highlights both established and relatively new migrants, offering insights into different stages of urban integration.

Educational attainment was relatively high overall. Nearly half (48%, *n* = 11) had completed secondary education, while 30% (*n* = 7) held a university degree. A smaller proportion (9%, *n* = 2) reported only primary education. The presence of both highly educated and less-educated individuals allows for a more diverse perspective on urban living experiences.

Income distribution revealed that the 3,000–5,000 RMB range was the most common (47.83%, 11 individuals), aligning with Chengdu’s mid-range income levels. Whereas 21.74% (5 individuals) fell below 3,000 RMB, indicating some degree of economic vulnerability.

Taken together, the socio-demographic heterogeneity of the participants supports the analytical depth of this study. Differences in age, length of residence, and socioeconomic status helped surface distinct ways in which migratory elderly interpret park environments, thereby strengthening the credibility and transferability of the thematic findings.

### 4.2 Qualitative research results

The diversity of the sample was reflected in the thematic analysis. Age differences shaped the ways participants perceived restorative benefits—while younger seniors (50–65) emphasized physical activity and social interaction, the oldest-old often highlighted tranquility and spiritual connection. Women reported stronger safety concerns in dense vegetation. Length of residence also mattered: long-term migratory elderly described adaptation strategies and integration into local culture, whereas recent arrivals frequently expressed nostalgia and comparisons with their hometowns. Socioeconomic differences influenced expectations of park facilities, ranging from affordability concerns among lower-income participants to aesthetic and design preferences among higher-income groups.

**FIGURE 5:**
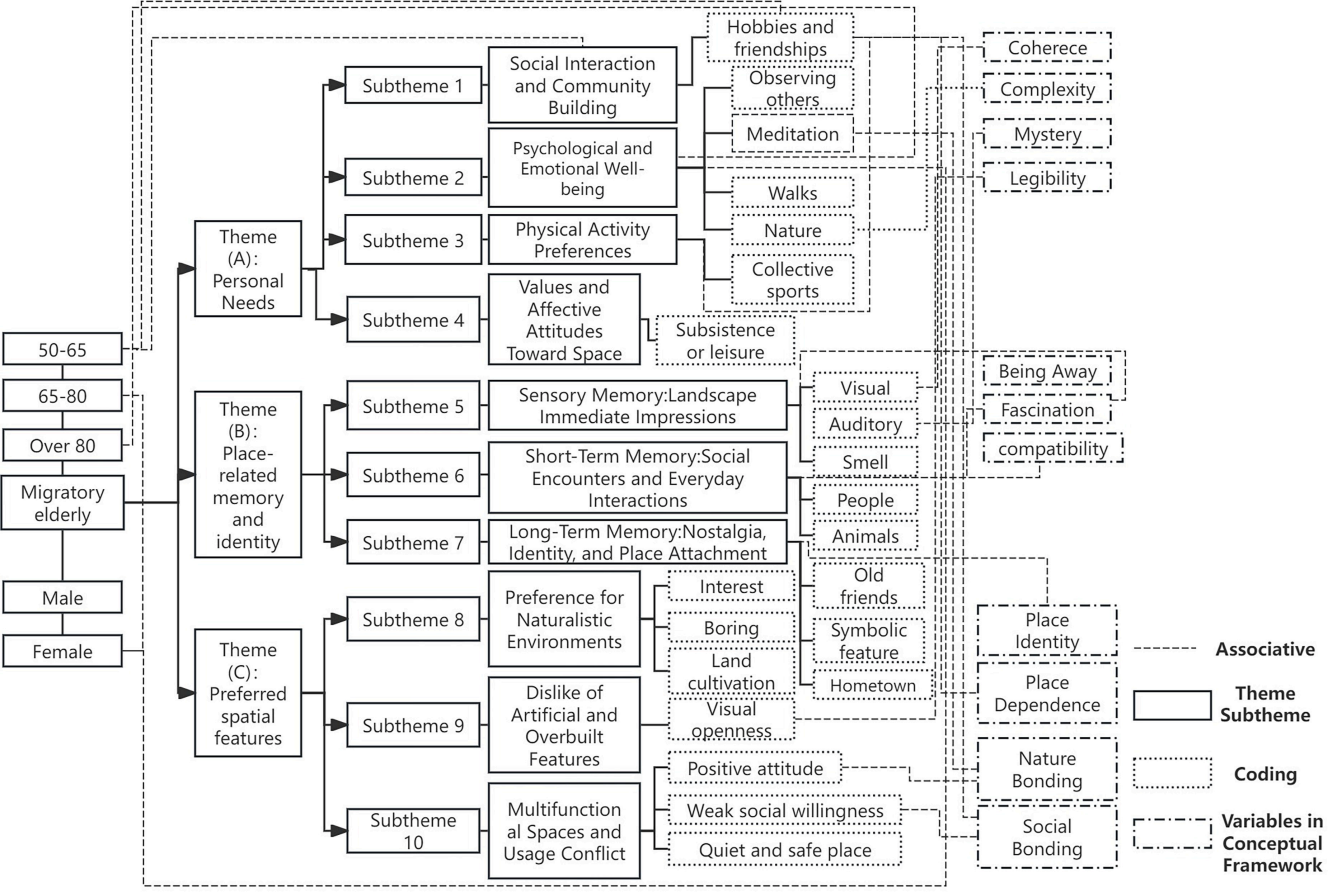
Coding tree for thematic analysis.

This iterative process resulted in the identification of three overarching themes that capture the restorative perceptions of migratory elderly in urban parks. A hierarchical coding tree was developed with three overarching themes—(A) Personal Needs, (B) Place-related Memory and Identity, and (C) Preferred Spatial Features—each with corresponding subthemes (see Figure 5). The Consolidated Standards for Reporting Qualitative Research (COREQ) standards were considered when reporting the data (Tong et al., 2007). A maintained codebook in Supplementary Material 4 defined each code with inclusion/exclusion criteria and exemplar quotations.

#### 4.2.1 Theme (A): personal needs

Parks afford restoration when they align with migrants’ needs for sociability, emotional regulation, bodily maintenance, and cultural continuity. Mismatch produces conflict or avoidance.

##### 4.2.1.1 Subtheme 1: social interaction and community building

Many respondents emphasized that shared hobbies—such as fishing, walking, and participating in group exercises—played a central role in forming casual friendships within park environments. Unlike enduring kinship or neighborhood ties, these relationships were typically forged through temporary encounters, positioning parks as informal yet vital social hubs for the migratory elderly. Lowering barriers to belonging in the absence of kin or long-standing neighborhood ties. This pattern suggests a context-specific social mechanism: routine, low-intensity co-presence in parks translates into “situational communities” that offer emotional support without the obligations of thick ties—valuable for migratory elderly who must manage care responsibilities and fluctuating time budgets. As one respondent explained:

*“Fishing suits people like us who can’t do strenuous exercise. It’s a light activity that helps adjust our mood, and we recognise the people we don’t know. We just met. When I came here, the people I met were my friends. We all have a common language.”* (P3)

Contrary to reports that walking may attenuate social interaction among older adults (Schmidt et al., 2019), post-meal walking in this Chinese sample catalyzed weak-tie formation and everyday conviviality.

*“Our neighbours came and got to know me. We got to know each other through joking and chatting. We often meet and go out for a walk together”.* (P23)

These findings suggest that urban parks are more than spaces for leisure or exercise; they also serve as platforms for social integration, emotional support, and community belonging among the migratory elderly.

##### 4.2.1.2 Subtheme 2: psychological and emotional wellbeing

Solitary practices (e.g., meditation) and indirect social observation (watching children) jointly supported mood regulation, underscoring the importance of quiet, natural spaces for introspection (Lee and Scott, 2017). At the same time, several participants highlighted the restorative value of indirect social observation, particularly of children and youth, which evoked feelings of vitality and continuity across generations (Uskoković, 2022). One participant explained:

*“I like to be by the river, watching children play. It improves my mood and connects me with my surroundings. When I sit here alone, time flies by. I enjoy the peace.”* (P8)

Nature itself emerged as a powerful source of psychological renewal. Participants often mentioned trees, water, and birds as central to recovery and even to “feeling young.” As another respondent described:

*“I prefer quiet places with trees and water. Parks should offer a feeling of being close to nature.”* (P9)

This echoes international evidence on the health benefits of natural environments for older populations (Lev et al., 2020) and aligns with Zhang and Zhang (2022), who emphasized the role of wild vegetation over designed flower borders in fostering place attachment. Landscape clarity and openness were also strongly linked to feelings of safety. Dense or obscured vegetation—often described as “deep” or “mysterious”—was associated with unease, especially among female participants. Participants described selecting benches beneath a layered canopy to avoid direct sun and scattered glare, especially near open plazas at midday.

“I always pick the spot under that big plane tree; the breeze there feels softer, and I can sit longer.” (P22)

Yet a minority prioritized visibility over shade due to perceived safety:

*“If bushes are too high, I’d rather sit in the open where I can see people coming.”* (P8)

These accounts illustrate the trade-off between microclimate comfort and situational awareness, shaping restorative use at different times of day. This resonates with research on gendered perceptions of safety in urban parks (Chen and Hedayati Marzbali, 2024; Polko and Kimic, 2022), as well as evidence that aesthetically appealing plant compositions tend to avoid heavy shrubbery (Tomitaka et al., 2021; Hoyle et al., 2017). Designing parks with clear sightlines, adequate lighting, and open layouts is therefore essential to support emotional security, particularly for elderly women.

##### 4.2.1.3 Subtheme 3: physical activity preferences

Respondents expressed diverse and sometimes conflicting preferences regarding collective sports, shaped by cultural background and tolerance for noise. While some valued the lively atmosphere of group activities, others perceived them as intrusive. One participant illustrated these cultural contrasts:

*“Elderly people in Sichuan may want to play mahjong and drink tea, but I prefer Tai Chi, singing, and dancing.”* (P15)

*“There are too many people doing square dancing. I don’t like it because I think it’s a bit noisy. I prefer quieter places.”* (P8)

Such divergent views highlight the need for parks to provide flexible activity zones that accommodate both energetic and tranquil forms of recreation. For migratory elderly in particular, physical activity preferences often reflect an effort to balance social connectedness, health limitations, and personal comfort.

##### 4.2.1.4 Subtheme 4: values and affective attitudes toward space

For many migratory elderly, attachment to urban parks was shaped not only by present-day preferences but also by historical experiences of agrarian life and food insecurity. References to “grain fields” and “food panic” indicate that for some, parks symbolically index security and subsistence, not only leisure. This explains why ornamental displays may be experienced as superficial, whereas “wild” vegetation signals authenticity and dignity. One participant recalled:

*“There is no need to improve parks. They are mainly grain fields. You have not experienced the food panic during that era.”* (P17)

These reflections suggest that environmental attitudes are deeply entangled with personal history and collective memory, echoing Tan et al. (2018) argument that creativity and awareness can foster strong place attachment regardless of length of residence. The findings reveal that for migratory elderly, enduring values and lived experience shape affective bonds as much as current sensory encounters.

#### 4.2.2 Theme (B): place-related memory and identity

This theme is examined through three interrelated perspectives—sensory memory, short-term memory, and long-term memory—drawing on Atkinson and Shiffrin (1968) model.

##### 4.2.2.1 Subtheme 5: sensory memory: immediate impressions of the landscape

Initial impressions of park landscapes were critical in shaping their willingness to explore and engage. Layered compositions (canopy–understory–water) drew people in and prompted exploratory movement. In ART theory (Kaplan, 1995), immediate sensory richness provides soft fascination that initiates attentional recovery. As one participant explained:

*“I like it when the landscape has different layers—like tall trees, flowers underneath, and water nearby. It makes me want to walk around and see more.”* (P12)

These findings suggest that sensory richness acts as both an immediate source of comfort and a stimulus for exploration, operating as a gateway to deeper emotional connections.

##### 4.2.2.2 Subtheme 6: short-term memory: social encounters and everyday interactions

Short-term memory in park contexts was frequently associated with recent social interactions with people and animals. Notably, gender differences emerged in patterns of socialization. Several respondents suggested that women were generally more proactive in initiating conversations and sustaining friendships:

*”I think women do have an advantage in making new friends. They are more willing to take the initiative to communicate, and it is easier to find common topics.”* (P4)

This observation is consistent with international research. For example, studies in the United States reveal significant gender-based differences in park use for physical activity (Pérez-Tejera et al., 2018). In addition, inclusive park features—such as all-age-friendly designs and pet-friendly facilities—were regarded as important enablers of social connection. Respondents highlighted that casual encounters—often facilitated by shared hobbies or pet ownership—helped them forge new relationships and strengthened their sense of belonging to the space. For some participants, emotional attachment was closely tied to these everyday encounters. One participant reflected:

*”I have a special attachment to this park because I found my dog here five years ago. It’s a significant memory.”* (P8)

The short-term memories provide ongoing social support, strengthening positive emotion, buffering stress, and contributing to cognitive recovery beyond immediate sensory effects.

##### 4.2.2.3 Subtheme 7: long-term memory: nostalgia, identity, and place attachment

Long-term memory among migratory elderly participants frequently revolved around nostalgic recollections of hometowns and past friendships, which were reactivated within their new urban environments. Such memories not only preserved continuity of personal history but also served as emotional bridges linking past and present, thereby reinforcing place attachment. As one respondent reflected:

*”Although I have lived here for many years, I often think of my old friends and my hometown in this park. These memories make me feel somehow connected to this new place as well.”* (P10)

Participants expressed a strong preference for spaces that evoked cultural or historical resonance—such as museums, memorials, or heritage-themed environments—which they perceived as emotionally restorative. For example:

*“Anyway, at noon, I went to look at the exhibits. All the unpleasantness was forgotten, and I felt so relaxed.”* (P2)

Specific landscape features also functioned as memory triggers. References to “canals” and “windmills” recalled participants’ hometowns, while one individual explained:

*“Seeing water wheels here reminds me of my hometown and brings back memories of my childhood.”* (P22)

These reflections align with prior findings that historically or symbolically rich landscapes enhance emotional bonds with place (Tan et al., 2018). These reflections align with prior findings that historically or symbolically rich landscapes enhance emotional bonds with place. As one participant noted:

*“I have feelings for it when I look at it every day. If it is replaced with tropical branches, the atmosphere will be different, but over time, I adapt.”* (P3)

Within the framework of restorative cognition, these deep memory-based attachments provide existential security and a sense of coherence, thereby enhancing the level of psychological restoration.

These subthemes highlight the layered role of memory—spanning sensory impressions, everyday interactions, and nostalgic recollections—in shaping elderly migrants’ emotional bonds with urban parks. By triggering multiple levels of memory, parks function as restorative spaces: sensory memory supports attentional recovery, short-term memory reinforces social connectedness and emotional stability, and long-term memory ensures identity continuity and existential wellbeing. In this way, place-related memories not only enrich personal meaning but also strengthen the restorative potential of urban parks for migratory elderly populations.

Overall, the evidence suggests that urban parks serve as mnemonic landscapes, connecting different stages of life and sustaining identity through place-related memories—a phenomenon observed across global contexts (Rishbeth and Powell, 2013). Consistent with quantitative links between memory, attachment, and restorativeness (Ratcliffe and Korpela, 2018) and with the view of landscapes as symbolic carriers of identity (Scannell and Gifford, 2010a).

Sensory memory corresponds to the fascination phase of the restorative process, where visual and auditory harmony gently captures attention and initiates cognitive renewal. At this short-term stage, memory anchored in everyday social events sustains the “being-away” and compatibility phases of restoration, where interaction and small rituals transform brief encounters into a restorative experience. At this long-term stage, autobiographical and identity-based memories close the restorative loop by anchoring self-continuity and meaning—transforming temporary comfort into enduring wellbeing.

#### 4.2.3 Theme (C): preferred spatial features

##### 4.2.3.1 Subtheme 8: preference for naturalistic environments

Jahani and Saffariha (2020) argue that urban park landscapes with minimal built structures are more effective in alleviating citizens’ mental stress. Similarly, individuals with a strong affinity for natural settings report enhanced restorative benefits (Wilkie and Clouston, 2015). Consistent with these findings, migratory elderly participants in this study expressed a clear preference for naturalistic environments, perceiving them as the most restorative. One participant described the emotional comfort derived from riverside landscapes:

*“When I walk by the river, I feel calm and relaxed. The sound of the water makes me forget the worries in my heart.”* (P6)

Follow-up interviews also revealed a general dislike for artificial landscapes, particularly built structures. As one participant noted,

*”Smaller buildings are fine. I think if you have too many buildings in the park, you probably can’t reflect the nature of the park.*” (P2)

Notably, for migratory elderly, these features also resonate with agrarian identities, not only with universal biophilic tendencies.

##### 4.2.3.2 Subtheme 9: dislike of artificial and overbuilt features

Participants consistently emphasized their preference for nature or open spaces such as rivers, lakes, and open courtyards. This echoes Subiza-Pérez et al. (2020), who observed that open squares hold therapeutic value for older adults. While squares were valued for facilitating social encounters, their restorative potential diminished when they became overly constructed or visually cluttered.

*“I like sitting in the courtyard under the trees. If you put too many walls or buildings, it feels closed and heavy.”* (P9)

Visual openness was described as essential for psychological relief:

*“When I look out and see water and open sky, I feel light. But if there’s too much concrete, it’s like the park is losing its soul.”* (P12)

These accounts suggest that excessive architectural interventions may compromise both restorative perceptions and emotional wellbeing by obstructing openness and natural views.

##### 4.2.3.3 Subtheme 10: multifunctional spaces and usage conflict

Open spaces that support diverse activities were widely appreciated for promoting social interaction; however, participants also highlighted the tensions that arise when quiet leisure is disrupted by noisy group events.

*“I like to chat with friends by the lake, but sometimes the music from dancing groups is too loud, and we can’t hear each other.”* (P4)

Others called for differentiated spatial zones to accommodate varied needs:

*“Different people have different needs. I think the park should have separate places—some for quiet, some for lively activities—so everyone can enjoy.”* (P7)

These observations point to the dual role of multifunctional spaces: while they facilitate inclusivity and social bonding, they also require careful planning to minimize conflicts and safeguard restorative experiences.

Across all subthemes, natural elements emerged as central to shaping restorative perceptions among migratory elderly individuals. Built structures, while generally offering limited direct restorative value, could enhance psychological benefits when carefully integrated with natural settings and designed to support socially compatible uses. The findings underscore the need for spatial planning that balances naturalness, openness, and multifunctionality, ensuring that parks not only accommodate diverse activities but also sustain their restorative potential.

## 5 Discussion

Taken together, the findings suggest a three-dimensional framework of migratory elderly park-use: encompassing needs, memory and cultural identity, preferred spatial features. These dimensions interact to shape restorative experiences in urban parks. By revealing how mobility and cultural continuity shape restorative perceptions, this study extends restorative environment theory beyond stable residential contexts.

Compared with findings from Western contexts—where later-life migration is often motivated by lifestyle preferences or amenity-seeking aspirations (Bender et al., 2020)—the present study reveals a more family- and health-oriented migration logic. Chinese migratory elderly tend to develop emotional bonds with places that facilitate intergenerational support and everyday caregiving. In contrast, research in Europe, such as in the Netherlands, emphasizes social integration and neighborhood familiarity as key contributors to migrants’ wellbeing (Hussein et al., 2024). Within the Chinese context, however, the restorative potential of parks is anchored in functional accessibility, intergenerational interaction, and the continuity of cultural identity, rather than purely aesthetic or affective experiences.

Accordingly, the proposed framework situates place attachment within a non-Western, mobility-affected aging population and demonstrates that restorative experiences are culturally embedded processes shaped by collective values and migration trajectories (Land cultivation complex), rather than solely by individual preference. This perspective refines and contextualizes theoretical understanding of restorative place-making across diverse cultural settings and suggests that existing models of place attachment should adapt to incorporate functional, natural, and social-bonding dimensions.

### 5.1 Preferred spatial features

Building on the results of Theme C, which revealed participants’ pronounced preference for nature-dominated environments, with nuanced differences in how individuals engaged these settings.

Preferences for both solitary and social activities highlight the heterogeneity of the migratory elderly’s restorative practices. For some, urban parks offer places for quiet reflection and emotional release. For others, they serve as venues for lively collective activities such as dancing or group exercise. This variation reflects not only diverse lifestyles but also deeper cultural expectations and personal histories of land-based practices. It underscores that restorative experiences are plural rather thanuniform.

In line with previous scholarship on the restorative capacity of nature-rich environments (Loukaitou-Sideris et al., 2016), this study demonstrates that natural elements—particularly vegetation, water features, and opportunities for hands-on engagement such as gardening—play a pivotal role in fostering both psychological restoration and place attachment. Importantly, these associations extend beyond aesthetic appreciation to reflect enduring agrarian traditions, value systems of self-sufficiency, and symbolic ties to the homeland.

By contrast, ornamental or overly structured architectural features were generally viewed less favorably, especially when they obstructed openness or limited contact with nature. However, participants also recognized the necessity of certain built elements when designed in ways that support compatibility between activities and enhance, rather than diminish, the natural atmosphere. This highlights the challenge and opportunity of park design: to preserve ecological and symbolic qualities while also accommodating diverse activity needs. Successful inclusion strategies are based on alignment between private actors’ initiatives and public actions (De Haas et al., 2021). Therefore, the findings point to the importance of inclusive and flexible design strategies that integrate naturalistic settings with thoughtfully designated zones, ensuring that quiet contemplation and vibrant social engagement coexist harmoniously.

### 5.2 Restorative perception factors

While spatial preferences highlight the tangible features of parks that attract migratory elderly, the underlying restorative perceptions are shaped by deeper psychological and cultural processes. The inclination toward naturalistic environments is not merely an aesthetic choice; rather, it reflects pathways through which individuals seek emotional comfort, continuity, and identity reconstruction in unfamiliar urban contexts.

This study extends existing theories of restorative environments by emphasizing the role of memory and nostalgia in shaping the experiences of the migratory elderly. For this group, emotional comfort is often anchored in environmental cues that evoke their hometowns. These cues function as active restorative mechanisms, stabilizing psychological states and reinforcing cultural continuity despite geographic displacement (Di Masso et al., 2019).

Traditional agricultural features—such as small vegetable plots—and native plant species were frequently mentioned as triggers of familiarity and agency. These elements enable migratory elderly to re-establish continuity across places, addressing both psychological and cultural needs that transcend conventional park design. In contrast, many non-migratory elderly populations tend to prioritize recreational infrastructure over memory-laden or symbolic landscapes, underscoring the distinctive restorative logics at play for migrants.

Moreover, the findings reveal a dual attachment pattern: nostalgic longing for homeland landscapes coexists with a gradually developing bond to current green spaces. This duality illustrates the fluid nature of identity, where past and present geographies intersect to sustain a coherent self-concept across transitions (Bazrafshan et al., 2021; Kasemets and Palang, 2023). Collectively, these insights suggest that incorporating familiar cultural and ecological references—such as native flora, small-scale agricultural areas, or historically resonant spatial patterns—can strengthen emotional bonds with urban parks and enhance their restorative capacity for migratory elderly populations.

### 5.3 Design recommendation

Integrating insights from sections 5.1 to 5.2, this section translates the empirical findings into actionable guidance for each stage of the urban park design process (Table 2). Designing restorative and inclusive green spaces for migratory elderly requires embedding emotional, cultural, and sensory dimensions into every design phase, from planning to post-occupancy evaluation.

**TABLE 2:** Design stages and policy strategies informed by findings.

Design stage	Key strategies informed by findings
Planning and conceptual design	Involve the migratory elderly through participatory workshops to identify emotionally resonant features (e.g., native vegetation, hometown motifs, small garden plots). Establish a multi-functional framework that balances quiet restorative areas with active social zones.
Spatial layout and zoning	Design a clear spatial hierarchy separating tranquil zones (woodlands, water features) from activity zones (square dancing, tai chi), using vegetation buffers to manage sound and visual intrusion. Ensure barrier-free circulation and intuitive pathways connecting restorative nature and social nodes.
Facilities and infrastructure	Provide shaded seating, accessible toilets, pavilions, and lighting to ensure comfort and safety, and also facilitate spontaneous social interactions. Avoid excessive hardscaping; use natural materials and forms that blend with vegetation. Incorporate sensory features such as aromatic plants, seasonal blossoms, and birdsong to enhance biophilic connection.
Programing and social management	Support both solitary and group use through flexible activity planning. Initiatives such as community gardening or intergenerational programs foster belonging, identity continuity, and light physical activity, such as native flora, small gardening plots, or familiar rural landscape motifs.
Maintenance and evaluation	Implement feedback systems (e.g., user surveys, community liaison groups) to monitor satisfaction and adapt design interventions. Encourage local stewardship to maintain ecological and cultural authenticity.

Naturalness functions as a dual driver that is both biophilic and culturally grounded. Prioritizing combinations of vegetation and water, and adopting structured naturalness with layered canopies, open understory, and accessible water edges, delivers soft fascination while maintaining safety. Openness and viewshed integrity should be preserved. Maintaining long, unobstructed vistas to water and sky reduces vigilance and enhances psychological lightness, while vertical clutter and enclosing walls should be avoided in primary gathering areas. Multifunctionality requires deliberate conflict management. High-conflict activities should be separated in space or time, for example, by locating amplified dancing away from lakeside conversation zones or scheduling peak uses to minimize overlap. Inclusive micro-affordances strengthen social ties and memory. Providing pet stations, all-age seating clusters, and small exhibit niches catalyzes positive micro-interactions and memory formation, thereby reinforcing repeat visitation and place attachment. Integrate native vegetation, agrarian water features, and modest heritage cues that evoke familiarity without resorting to pastiche; Surface locally meaningful symbols.

**FIGURE 6:**
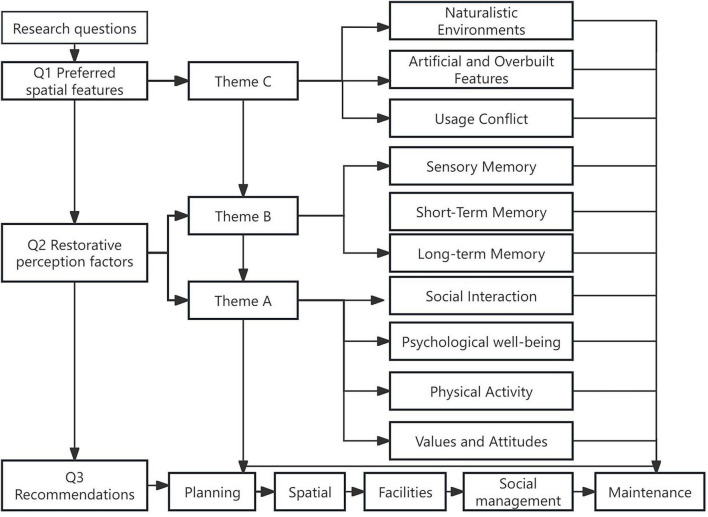
Main finding.

In doing so, park design can transform urban green spaces from mere sites of recreation into meaningful landscapes of belonging, thereby advancing both restorative benefits and the overall wellbeing of migratory elderly populations. Figure 6 links the research questions with the thematic findings and the resulting recommended policy levers, highlighting how preferred spatial characteristics and restorative perceptions are translated into practical recommendations.

### 5.4 Policy and societal implications

China’s rapid population aging and internal migration create pressing demands for inclusive, age- and mobility-sensitive urban green spaces. First, restorative design should be embedded in urban policy frameworks. Incorporate restorative and cultural dimensions into park planning standards alongside ecological and functional goals, aligning with Healthy China 2030 and Age-Friendly City initiatives. Second, cross-sector collaboration among planners, landscape architects, social workers, and community organizations is crucial for translating restorative principles into practice and sustaining community wellbeing. Third, local pilot projects and adaptive governance can operationalize these ideas. Municipalities should test participatory and culturally informed park designs, using feedback loops between users and planners to refine management strategies. Finally, the proposed framework has broader global relevance. In rapidly urbanizing regions across Asia, Africa, and Latin America, where aging and mobility intersect, integrating cultural memory, mobility history, and biophilic design can enhance both wellbeing and social cohesion.

Building on classical restorative environment models, the proposed framework repositions restorativeness within contexts of mobility and cultural adaptation, thus expanding its theoretical boundary.

## 6 Conclusion

While basic amenities remain essential for all elderly populations, migratory elderly may particularly benefit from targeted interventions such as community gardening areas, biophilic features rooted in their regions of origin, and culturally embedded visual design. Design implications include the need for inclusive and multifunctional parks that combine quiet, nature-oriented zones with spaces for group activities. Integrating biophilic and culturally resonant elements—such as native vegetation, familiar flora, and small pavilions—can strengthen belonging and restorative engagement.

Beyond practical design considerations, this study advances theoretical understanding by introducing a migration-linked restorative framework that explains how mobility and cultural reconstruction shape restorative perception. Compared with their Western counterparts, Chinese migratory elderly display stronger intergenerational and functional orientations, reflecting family-centered motives and collective wellbeing embedded in Chinese culture.

The insights contribute to global debates on aging, migration, and urbanization by revealing how relocation reshapes environmental well-being. Nevertheless, the study’s regional scope and convenience sampling limit its generalizability, as participants may disproportionately represent socially active individuals with stronger park engagement. The gender composition of the research team may also have subtly influenced participant interactions and merits further reflection. Moreover, because the sample consisted primarily of urban residents, rural migratory elderly—whose mobility motivations and patterns of park use may differ substantially—remain under-represented.

Despite these limitations, the findings enrich international discussions on aging and urbanization by demonstrating how migration-related discontinuities reconfigure place-based wellbeing. The proposed framework may also hold relevance for other rapidly urbanizing societies in Asia, Africa, and Latin America, where demographic aging intersects with high internal mobility. Future research should refine this framework through cross-cultural and cross-city comparisons, exploring how intergenerational memory, perceived safety, social cohesion, and environmental familiarity shape the restorative potential of green spaces. Such inquiries can build a more nuanced evidence base for designing inclusive and culturally attuned urban landscapes that meet the evolving needs of aging populations worldwide.

## Data Availability

The original contributions presented in the study are included in the article/Supplementary material, further inquiries can be directed to the corresponding author.
