# Supplementary material for: Restorative perceptions of migratory elderly: insights for inclusive urban park design in china
Source: Front Psychol. 2025 Nov 28;16:1695092. doi: 10.3389/fpsyg.2025.1695092 (PMC12698403; doi:10.3389/fpsyg.2025.1695092)
Supplement: Supplementary file 1 [file Data_Sheet_1.pdf]

Supplementary Material-04

Contents

1 Supplementary Data 2

1.1 Interview Question (for Qualitative Data) 2

1.2 Coding Details 5

1.3 Interview Contents Selected from Original Data 11

1.4 Data Coding Process Example 20

2 Supplementary Figures 25

1 Supplementary Data

1.1 Interview Question (for Qualitative Data)

I. Interviewees: Non-local residents over 50 years old in Chengdu's urban parks.

II. Interview format: Semi-structured in-depth personal interview completed independently after visiting the park. Requirements for the results: audio recording, taking photos, and converting them into text for submission. (15 sets of documents, each containing audio, photos, and words)

IV. Notes:

1. Before the interviews began, the researcher explained that there was no right or wrong answer and that the participants were regarded as "experts" and were encouraged to speak freely.

2. Participants wore tape recorders to record the interviews and were reminded that all the information they provided was strictly confidential. 3. Recording avoids the distraction of paper and pencil notes and better focuses the participant's attention.

V. Procedural process:

1. Greeting: The researcher introduced herself, the purpose of the study, and the value of park enhancement.

2. Anonymity: The confidentiality procedure and the conditions of anonymity of the subjects were emphasised.

3. Interview: Two consecutive sections.

1) **Structured Self-Report Survey:** subjects were asked to complete a short (approximately 5-minute) structured self-report survey to describe their demographic characteristics and visitation behaviour (Table 1).

Supplementary Table S1. Demographic Characteristics and Visiting Behaviour

	Notes(If it is optional, please tick)
Study site：	
Time of living here：	
Age：	
Gender：	

Income per month:	
Highest education:	
Frequency and time spent:	
Activities in parks:	
Coming alone or with someone else:	Yes, expand on attending social events in the park:<br>a) Have met new friends from the park<br>b) have met friends already<br>c) With family
	No. Generally:<br>a) alone<br>b) with grandchildren<br>c) Walking the dog

2) Semi-structured in-depth personal interviews: the interviews lasted 20-40 minutes, and the interview questions were posed in everyday language understood by the respondents. Five topics were included, and four steps were used for each topic.

Four steps:

- l Introductory introduction: what can you tell me about ***?
- l Exploratory question: Can you tell me a little bit more about the experiential view of ****? Why do you think ***?
- l Specific question: Can you give me an example?
- l Concluding question: thank you, followed by the next topic.

Five topics:

Topics 1-4 are structured around structured topics. l Topic 1 gathers information about the emotions, feelings, and retrospective reflections that participants evoked in the park by asking them what they liked or disliked about the park. l Topic 2 is a summary question that asks participants what they liked or disliked about the park. Topics 2-4 guided participants to trace landscape elements, attractions, people, and

activities to explore their preferences, restorative experiences, and level of attachment to the park. Topic 5 concludes with open-ended questions and acknowledgements.

- Topic **1: Where do you feel you like to be in the park the most, and where do you like to be the least? Or, which of the following spaces felt most restorative? Tell us why.**

"Meadows, woodlands, neighbouring rivers, neighbouring lakes, squares, buildings, dams enclosed by buildings, wide paths, trails" are the most likely things to do in a park; physical activities and social interactions (meeting with others) in a park.

- **Topic 2: What qualities of the park do you prefer? Which features make you feel restored?**

✧ The scenery is layered and neat, continuous and repetitive.
✧ The scenery is recognisable, not easy to get lost, and well signposted.
✧ The landscape is complex, unorganised, and varied.
✧ The scenery is attractive to explore further, the scenery is torturous, the scenery is deep and mysterious, and the scenery is novel.
✧ Participants can record these experiences and perceptions as carefully and comprehensively as possible.

- **Topic 3: What is closer to your feeling of recovery?**

✧ A sense of escape from the everyday (an experience of detachment from the mundane, relaxation of tense nerves, forgetting everyday constraints)
✧ Intriguing to get lost in (attractive qualities to explore further, very glamorous)
✧ The scenery is calming (enjoying what you like, being at one with the park, enjoying doing things in harmony with the environment)

- Topic **4: What is the level of attachment to this park? Going further, which of the following four statements is more agreeable, and which feeling is less obvious: Does identification with the park help to increase the experience of recovery? Do deeper feelings of attachment to the park increase the recovery experience? Do social connections at the park help increase the recovery experience? Promoting your connection with nature in the park helps to increase the recovery experience.**

✧ Sense of identity (frequenting is very emotional, have good memories, miss it when I leave, think of my identity and heritage, think of my loved ones, parents, children spouses)
✧ Sense of dependence (good for physical activity, safe from natural disasters, like living nearby, clean and good environment, refreshed, affects health and well-being, think this place should be protected at all costs, activities here are more meaningful than elsewhere)
✧ Social interaction (living near here is good for family bonding, helps me to meet more people in the community, I have made new friends here, there are a lot of community activities here, fosters a sense of belonging to the community)

✧ Nature experience (I feel at one with nature, I don't miss the local flora and fauna so much if they disappear, I know myself better here, and I feel happy here)

- **Topic 5: Ideal or perfect park in mind.Whether they have visited other parks and why; park features (social and natural) that might encourage or discourage them from visiting the park; suggested changes to the park that would make them more inclined to visit the park/do physical activities/spend time with others;**

1.2 Coding Details

Supplementary Table S2.Axial coding based on the open coding

Axis Coding	Open coding
Explore the unknown; Clear; Sunshine; Too artificial is boring	Those who haven't walked yet should go; Clear and not lost. The deep scenery is uncomfortable; I like sunny places; Neatness is boring; No need for human intervention.
Layered;Colorful<br><br>Regular;Designed	There are views all the way; The scenery is varied and the colours are rich; Well planned and neat.
Feel safe; Educated<br><br>Not easy to get lost	Road clean; Feel dependence; Fear of getting lost; Scientific knowledge propagation; Bright light; Have open spaces.
Unexpected design;<br><br>Clear at a glance	Plants are layered; not too neat; not repetitive; unexpected design; Clear at a glance; and not too complicated.
Memory;Refresh;Daily Habits	Digestion requires walking; Family communication; Quiet and not noisy (no square dancing); Feeling young again; Running feels integrated; There are memories (picking up a puppy)
Similar scenery (tree branches); History (waterwheel); Companions (friends,	Similar scenery brings back memories; No hardship in the countryside, just having fun; I would want to play in the park with my neighbours when I went back to my hometown; I work hard in my hometown to exercise my

dogs, myself);<br><br>I miss home more. Grow crops in the countryside to exercise; Want to play with neighbours in the park when I go back home; Rural life is free.<br><br>The land is wasted; Food panic is satisfied.	body and grow crops.<br><br>Rural daily necessities are free of charge; Parkland is not used to grow crops and is overgrown with weeds; It is better to grow vegetables than to have empty land; Food panic; I hope it's like a forest, it's just a place to kill time with children.<br><br>Reminiscent of memories from different stages; There is no emotion, just wasting time, and I miss my hometown more; It's nice to have a waterwheel in the park, which is only found in rural areas; I might think of it when I go to a park outside; I would think of it because I am always with my dog; Idle thinking about myself and life; The city is humanized; History brings back memories: development is not easy; The shape of the branches is different, compared with the past tropical.
Light socialisation;<br><br>Light physical<br><br>Activity	Likes diverse crowdsnot noisy; Don't like commercialization; Promote family harmony(A good mood); Meet someone you know; Square dancing is too noisy; Photo exhibition brings back memories; Chrysanthemum exhibition attracts photography enthusiasts; Chat with neighbours and family; Play cards; Bring grandchildren to find similar; Dancing makes people feel younger; Chatting makes people forget their worries; Didn't know each other a year ago, and made new friends in the park; Too many buildings and too many artificial traces; Take photos and share them on social media; Gathered together because of similar interests; I didn't meet anyone new (because of my personality); Not social (short walks to relax); The time for meeting friends is not suitable (chance social encounter); Don't like strangers, but I can have tea with friends; I like watching kids playing in the water.
Waterscape;Colorful plants; Smellscape<br><br>Soundscape	Plants near lakeside; Get used to green; The sound of water winds, and birds; The smell of grass; The colour of flowers; Cool under the tree; The desire to share flowers; Sad tree being cut down; Knowing plants; Peaceful, Come back to life; Terrain changes;
Humanities and history; Recorder; Flowers fragrance; Away with the chores	Get away from the chores; The air and greenery make you relax; Popular science of humanities and history makes people escape from the world; Sleeping in a daze; The fragrance of flowers reminds you of faraway places; Time becomes your own; The city is too noisy; Figure out the troublesome things that can't be figured out on the sofa; The recorder sounds melodious;

Novelty; Primitive<br><br>Not too new	Change the scenery with every step; Novelty is more interesting; Too new, no nostalgia; favourite primitive landscape; Best terrain changes; I want to go to a place I haven't been to before.
Activity area; Stay area<br><br>; Walking area	Walking on the quiet path; Diabetes must walk; Close your eyes and walk safely and relax; Sitting and thinking; Watching children playing in the water; watching teenagers playing ball; The sound of windmills and water reminds me of my hometown; Listening to opera and drinking tea; Take pictures and show them to friends back home; Playing Mahjong and Drinking Tea; The leaves are wide and tall, dancing in a large place; Bring favourite food; Warm atmosphere, lying on the grass;

Supplementary Table S3. Three parts of open coding were devised from the interview section

Theme	Details							
1. Needs	Hobbies and friendships	Observing others	Solitary meditation	Digestive walks	Experiencing nature	Collective sports	Grandparents	Subsistence or leisure
	Subtheme 1: Social Interaction and Community Building	Subtheme 2: Social Interaction and Community Building				Subtheme 3: Physical Activity Preferences		Subtheme 4: Values and Affective Attitudes Toward Space
Content	Light social interaction and light physical activities	Multiple people to get the reflection	Relationship with Compatibility and Nature	Partly associated with social interaction	Sound, smell, vision	Clear love and hate	Don't be bored	Popular without too many buildings
Refer to the appendix.	(P3, position 145-150)(P3, position 59-61)(P23, ranking 37)	(P5, location 88) (P4, location 135-144) (P13, location	(P11, location 79-99) (P8, position 120-122)<br><br>(P10,	(P9, position 113) (P22, position 182) (P2, position 31-34) (P23, location 359-	(P10, location 22-23) (P9, position 65-67)(P3,	(P19, position 176) (P19, position 164) (P6-4, position 45) (P7, position 288) (P10, position 65-66) (P10, position	(P16, position4-5) (P8, position 182) (P13, location	(P22, position 162) (P7, location 329-331) (P23, position 49) (P23, location 216-217) (P7, location 339-341)

		578-588) (P13, location 498-506) (P8, location 105)	position 30-32)	363)<br><br>(P9, position 65-67) (P3, position 118-120)	position 118-120) (P9, position 97)	62-63) (P4, ranking 114-120) (P20, ranking 32) (P15, position 12)	344-360)	
2. Place-related memory and identity	Sensory register			Short-term memory		long-term memory		
Content	Visual	Auditory	Smell	People	Animals	Symbolic feature	Hometow n	Old friends
Refer to the appendix.	(P23, position 83-85) (P3, position 159)	(P10, position 50)	(P19, position 80)	(P12, position 30-37) (P19, position 44) (P23, position 363) (P17, position 11-14) (P22, location 266) (P10, position 39)	(P8, position 134-141)	(P9, position 141-142)(P4, position 79) (P3, position 157) (P11, location 33-41);(P4, position 185-187);(P16, location 25-27); (P4, position 185)	(P11, position 170-179) (P4, position 68-76) (P9, position 115)	(P11, position 170-179)

<table>
<tr><td rowspan="2">3. Preferred spatial spaces</td><td colspan="4">Affect</td><td colspan="3">Behaviour</td><td colspan="3">Cognition</td></tr>
<tr><td colspan="4">Preference for Naturalistic Environments</td><td colspan="3">Multifunctional Spaces and Usage Conflict</td><td colspan="3">Dislike of Artificial and Overbuilt Features</td></tr>
<tr><td>Content</td><td colspan="2">Boring</td><td colspan="2">Interest</td><td>Physical activities</td><td colspan="2">Social interaction</td><td>Positive attitude</td><td colspan="2">Weak social willingness</td></tr>
<tr><td>Refer to the appendix.</td><td colspan="2">(P13, position 400-408)</td><td colspan="2">(P23, position 403-404)</td><td>(P8, position 62)</td><td colspan="2">(P16, location 25-27)</td><td>(P4, position 102-107)</td><td colspan="2">(P9, position 123) (P3, position 45-46)(P4, position 86-87) (P7, position 180) (P8, position 40-41) (P9, position 60)</td></tr>
<tr><td rowspan="2">Content</td><td colspan="4">Land cultivation</td><td colspan="3">Visual openness</td><td colspan="3">Quiet and safe place</td></tr>
<tr><td>Food panic</td><td colspan="2">Livin g cost</td><td>Food safety</td><td colspan="2">Rich in plants</td><td>Unexpected</td><td colspan="2">Quiet and varied people</td><td>Legibility</td></tr>
<tr><td>Refer to the appendix.</td><td>(P17, position 27) (P22, location 310) (P22, position 306)</td><td colspan="2">(P22, locati on 202)</td><td>(P22, positi on 198)</td><td colspan="2">(P19, position 411) (P15, position 18)(P19, location 70) (P22, position 270) (P10, location 25)(P13, position 386-390)(P9, position 129) (P9, position 67)(P3, position 96-97)</td><td>(P13, position 386-390)(P9, position 129) .</td><td colspan="2">(P9, position 67)(P3, position 96-97)</td><td>(P9, position 75)(P8, position 81-86) (P4, position 41-42)(P4, position 37)(P23, position 89) (P8, position 78-79) (P2, position 38-44)</td></tr>
</table>

1.3 Interview Contents Selected from Original Data

Personal Needs

(P3, position 46-48) "Now it's like this, I ask him out, he says he'll consider it, he asks me out, I say I have something to do, I just want to come out today casually, and when I go out, I meet new friends, I don't want to get together. We used to get together, but now we get together less and less. "

(P3, position 145-150) "Have you made some new friends here? Yes, when we fish here, we recognise all the people we don't know. We just met, when I came here, all the people I met here are my friends. We all have a common language, and I can communicate with them. You can see people from all over the country coming here; they all have a common language when they fish here. We all feel very satisfied."

(P3, position 59-61) " They usually met through fishing, but they didn't know each other in the past. They hang birds every day, they have to hang cages on branches, or they all contacted each other three blocks ago, and those pigeon friends were also temporary acquaintances. "

(P3, position 75-87) "Fishing is suitable for people like us who don’t exercise much. Some people can’t do strenuous exercise, so it’s a light exercise. But we young people can’t do strenuous exercise, so we just take a walk, walk slowly, and adjust our mood. It may mainly be walking along the river, where there are trees. Most people like to chat and fish by the lake to exercise, which is suitable for ordinary people, especially the elderly. No money is required. "

(P23, ranking 37) " I don’t like walking the dog, for example, they don’t pick up dog poop, and it’s uncomfortable to step on dog poop. I still like to walk on the lawn, which feels more comfortable. "

(P5, location 88) "Only here can they be seen fishing. In addition to observing the activities of the crowd, some migratory elderly people observe animals to understand themselves. "

(P4, location 135-144) "Why do I say that the environment in this place is good? Because there are birds, there are things in the water that eat fish, and there are birds that eat fish. This is why we take good care of it. Some people along the way are very caring, and many elderly people want to raise birds. They insist on getting up every morning and scattering food, millet, and corn on the platform for the birds. The birds will naturally come to the high platform. I said that these are all bird lovers. Because everyone cares so much about the environment, it is still good for our time. Otherwise, you would not know that the water is not good by looking at the birds. Birds can prove that the water is good, but the environment is not good. Isn't it just to understand yourself better, understand this society better, understand the government better, and understand the lives of people around you better? Or everyone's living conditions, the government. Society should say that people nowadays are very happy. What is the reason? People of my age have suffered since childhood and served in the military, so I cherish my current life very much. "

(P13, location 578-588) "It should be suitable for people who take their children out for a walk, people who walk their dogs, and young people who play table tennis and basketball. Anyway, it should be comprehensive and at least be able to integrate people of all ages. (P3, position 161) For

example, if we use it, it can only be used by people of our age group. However, there are many children or young people who come here, and they don't know what to do and may not be that interested. We still hope that this park can be open to both the elderly and young people and children. We hope to see more groups in the park. "

(P13, location 498-506) "I think it should be suitable for people of all ages to gather, or a place to play and relax, or a place for one person to be alone, or a place for a few people to think quietly, it should be rich, not like some parks, where only children can play, or only the elderly can dance in the square, which is too monotonous."

(P8, location 105) "I like the scenery to be more peaceful, to do what I like, to take my dog for a walk, and to watch the kids playing in the water by the river. Walking the dog, and what I like most is to go for a walk with friends, take a walk, and chat. When you are in a bad mood, having friends to chat with you will make you feel better. The park has beautiful scenery, flowers and birds. I like to be by the river because I can see fish. There are also some children catching tadpoles and small fish there. "

(P11, location 79-99) "Do you see the windmill? You can hear the sound of water flowing past here. I sit here in the morning when there are few people and can still hear the sound of water and birds. It feels like I am part of nature. There are fish in this fish pond. The feeling of man and nature, the rain and water, are indistinguishable. "

(P8, position 120-122) "Especially when I sit here alone, thinking about something, time flies by unknowingly. I like to be alone and think. I prefer quietness and being alone. "

(P10, position 30-32) "This place makes people feel younger because they often go to parks and get away from the city. One reason is that the streets in the city are very busy. When we get older, we just want a quiet place. There are benches by the river, by the bamboo forest, or deep in the trail where we can sit and rest. It is very comfortable. If we are tired, we will not feel tired soon after sitting. The park has more negative oxygen ions than the streets. It is also good for health care. It is good for the elderly to have pneumonia and allergies. "

(P9, position 113) "We visit the park purely when we have nothing to do, right? It's not called leisure, but it can be said that we have nothing to do. We come here when we have free time every day, and walk around in the park. If we have nothing to do after dinner, we will go out for a walk. It's not that we are too dependent on this park. "

(P22, position 182) "We all feel good when we go out to the parks every night. There is not much to do in the house. We just go out to do something and walk every day. "

(P2, position 31-34) "I don't like to participate in social activities. Because I usually go out for a short walk, I don't participate in these activities to relieve fatigue. I feel that I have walked a long way, especially to relax my eyes, so I always want to go for a walk. "

(P23, location 359-363) "Our neighbours came and got to know me. We got to know each other through joking and chatting. We often meet and go out for a walk together. "

(P9, position 65-67) "Generally, I prefer quiet places. It is more comfortable to walk on a tree-lined path. If it is a large square, it is not a park. Generally, I prefer wetland parks. Wetland parks have

water, are near rivers, or have lakes. Some of them feel better. It’s more convenient to think. Generally, it is a quiet place. Sometimes I talk to friends or think about some problems. It is quieter. I like water, so I prefer places with water. I always feel that there are mountains, water and trees, which is the feeling of nature. Since it is a park, there is a feeling of nature. "

(P3, position 118-120) "Everyone has different goals and needs. I also forget about some of my daily worries, such as work and life. I forget all of this, sometimes even myself, and I feel it’s still okay. "

(P9, position 97) "Maybe you walk around in a city park to think better. You can talk about your things, such as what troubles you encountered at work, or what you encountered. Maybe you sit on the sofa at home and sometimes feel that the more you think, the more confused you become. Maybe you can feel better while walking around, such as walking in the park, or thinking while walking. City parks are places where you can relax and think. They are places where you can think while walking because, after all, they are close to where you live, where you work, and where you live. They are not places where you can completely relax when you travel. "

(P10, location 22-23) "I like this place, the bird's singing forest is quite large, and sometimes you have to go in to take a look. There are many birds, such as peacocks. Hearing the sounds of birds makes me feel closer to nature. There are also football matches, where some of their football staff participate in some mutual competitions. Seeing young people makes me feel younger. I am drinking tea by the river. Hearing the sound of flowing water makes people feel more relaxed and calm because it feels like the body and mind are closer to nature. For example, there is another place that is a bit like us closing our eyes and resting in the deep mountains. You can hear the sound of water, and there are also many bird sounds, and there are also wild animals on the riverside. There is a saying that the white crane is a relatively quiet place. Tazishan Park is relatively quiet, so I still prefer Tazishan Park. "

(P9, position 65-67) "Generally, I prefer quiet places. It is more comfortable to walk on a tree-lined path. A park is a park. If it is a large square, it is not a park. Generally, I prefer wetland parks. Wetland parks have water, are near rivers, or have lakes. Some of them feel better. It is more convenient to think. Generally, it is a quiet place. Sometimes I talk to friends or think about some problems. It is quieter. I like water, so I prefer places with water. I always feel that there are mountains, water and trees, which is the feeling of nature. It is the feeling of nature. "

(P3, position 118-120) "Everyone has different goals and needs. I also forget about some of my daily worries, such as work and life. I forget all of this, sometimes even myself, and I feel it’s still okay. "

(P9, position 97) "Maybe you walk around in a city park to think better. You can talk about your things, such as what troubles you encountered at work, or what you encountered. Maybe you sit on the sofa at home and sometimes feel that the more you think, the more confused you become. Maybe you can feel better while walking around, such as walking in the park, or thinking while walking. City parks are places where you can relax and think. They are places where you can think while walking because, after all, they are close to where you live, where you work, and where you live. They are not places where you can completely relax when you travel. "

(P19, position 176) " Everyone has to dance together to be happy. If one person can't dance, then everyone should dance together. "

(P19, position 164) " Anyway, I don't feel old when I go out. I feel 10 or 20 years younger when I dance. "

(P6-4, position 45) " Dancing is the passion of young people. We are old and can't dance anymore. I am old and can't dance anymore. "

(P7, position 288) " It's a bit noisy when singing. The elderly sing and dance here. "

(P10, position 65-66) " It is recommended to reduce the square dance here as much as possible, and those who are willing to dance should turn down the volume and try not to disturb others. "

(P10, position 62-63) "There are also many people dancing in the square. Because we old people like quiet and peaceful places. It is noisy, there are a lot of people shopping, shouting, and loud percussion music, which has an impact on the hearts of the elderly, so we try not to go there. "

(P4, ranking 114-120) "The park must be quiet and peaceful, the square is likely to be noisy, 60 old ladies come to dance, what are you doing here? You have to separate from them. There is no noise in places with children. Why are they noisy? Children's voices are very nice, right? "

(P20, ranking 32) "I don't dance square dance, but I like music. Sometimes I like to play the harmonica and electronic keyboard, and sometimes I also make vibrato. "

(P15, position 12) "Elderly people in Sichuan may want to play mahjong and drink tea, right? Well, I prefer to do some exercise, such as Tai Chi, Ba Duan Jin, singing, and dancing. I like this kind of music. "

(P16, position4-5) "The most important thing is to have more activities. When taking care of children, you can participate in more activities so that you won't be bored. "

(P8, position 182) "Because you can communicate with your family, and you can talk with friends about anything that makes you unhappy, or chat with friends, and everyone will feel good, which will help them become more and more dependent."

(P13, location 344-360) "A relatively quiet place with good scenery. It would be best if it had a warm atmosphere. For example, you can be with a few good friends on the lawn, have your tent, eat, have a picnic, etc., but there will be a wide distance between everyone so that they don't affect each other. It would be best if the weather is good, not too hot, and there is sunshine. "

(P22, position 162) "Anyway, at noon, I went to look at the exhibits. All the unpleasantness was forgotten, and I felt so relaxed. "

(P7, location 329-331) "Which scenery do you think is the best in the park? The monument is well done, and the story of the photo there is also more colourful, and the ancient paintings and biographies are all well done. "

(P23, position 49) "Smaller buildings like this are fine. I think if you have too many buildings in the park, you probably can't reflect the nature of the park, right? Yes, okay. If you give more nature, it will feel better. "

(P23, location 216-217) "This kind of park outside is natural. You don't feel a strong sense of nature because the houses are very close to nature. "

(P7, location 339-341) "The scenery is the most beautiful when there are flowers. There are also a few museums. I saw a chrysanthemum exhibition in the pavilion at the entrance hall, which was also beautiful. I saw a lot of people taking pictures. "

Memory

(P23, position 83-85) "Interviewer: It still has a sense of hierarchy, but it is not repetitive, or there are more flowers and combinations, and there is some more variable scenery, such as when you walk here, there is a tree with colour. It belongs to the next tree, and in the next stage, it will turn into another colour. I think this is also good. "

(P3, position 159) "Next to me, there are shady trees, gurgling water, colourful flowers, and lush grass. This must be beautiful. "

(P10, position 50) "You often hear the sound of birds, the sound of the river, and the fragrance of flowers. After all, in the park, sometimes the plants also emit special scents, which will enhance the experience. "

(P19, position 80) "I like to go around and play behind this place. The air is a little fragrant, and the flowers are blooming, which is quite relaxing. "

(P12, position 30-37) "If you have made new friends, you usually hang out with them. In the morning, we sing and dance. I don't have pets. It's time for me to play by myself. Children are the responsibility of their parents. "

(P19, position 44) " There are many new friends. Some aunties are dancing and doing exercises. "

(P23, position 363) "We got to know each other when we met, and we often went out for a walk together. "

(P17, position 11-14) "Are you friends with the aunt next to you? Yes. Friends are simple, as long as you have good relationships. We met while walking in the park, just killing time. We have a common language, and we can chat. People's personalities are also very complicated. "

(P22, location 266) " I have to make a lot of friends. We came here a year ago. We didn't know each other at that time, but we wandered around in these parks and got to know a lot of these people. "

(P10, position 39) "People in the community can go out for a walk together to enhance mutual understanding. Of course, we can sit in the park and chat with others. We can also communicate with each other. For example, the elderly can talk about topics that everyone is concerned about. And

those who come with their relatives can also increase the affection between relatives. Anyway, I think we still have to come here often, so I still have a certain degree of attachment to the park. "

(P8, position 134-141) "That is a good memory of this park. Do you have any specific good memories, that is, have you experienced any good things? "

"Yes, I have some in this park. The most profound one is because of my puppy. I picked it up in this park and fed it for 5 years. One time, I was walking in the park with my friend, and then I saw a puppy. My friend and I went to play with it. Because I didn't know that no one wanted it at the time, we played with it. It kept following us. We didn't dare to take it away. We looked everywhere and searched everywhere. We were afraid that it was someone else's dog. People must be anxious if they lose their dogs. Then we waited for a long time, but no one came to look for it, so we took it away. I took it home, so I like this park more, and this is the most important point. "

Q: " Then I bring the puppy here every day, and he feels like he is back home. "

A: " The first thing I consider is to bring it here every day to see if anyone is looking for it here because when I picked it up, it was just a little big, it seemed to be just one month old, and I brought it here every day to see if its owner was looking for it. Later, I gradually got used to playing here, and it became a habit. I brought it here every day to play. I have fed it for 5 years since I picked it up, and I set its birthday to the day I picked it up. "

(P9, position 141-142) "There will be some humanistic design in it, such as opening a science corridor to introduce some things, such as natural animals and plants, and other popular science. Because people will read these things to improve their knowledge. So while relaxing, you can read some texts, some humanities. You can also improve your knowledge. "

(P4, position 79) "When I travelled, I felt that Chengdu was a very easy-going place. Life was pretty good, unlike other places where things were really expensive. "

(P3, position 157) " I have feelings for it when I look at it every day. For example, this section of ours is by the river, and it looks very natural. If it is replaced with tropical branches, tall tropical branches, the atmosphere of the environment will be different, you know? The atmosphere is different, and you may be able to use this thing to adapt. For example, when we went to the coastal areas of Shenzhen, they didn't have branches like ours. Yes, the branches there were all tropical trees from Hainan. The big ones were different from us. But after you stay here for a long time, you will slowly adapt to this environment and feel relaxed. "

(P11, position 170-179) "In other places, you can see water wheels in the countryside. Now, you can hardly see them anymore. You can only see them on TV, in movies, or in other literary works. But I think this has the characteristics of the water wheels in western Sichuan. It gives me a better feeling. It makes people feel that the water becomes alive through the water wheels, and they miss the old times. "

(P4, position 68-76) "I just feel comfortable sitting here, just like young people, right? I came back after I got old and retired. I was a soldier and moved around everywhere before, so I didn't have a fixed place to stay. I settled here after the earthquake. I didn't have a fixed place to stay before, so I

moved around everywhere. The good troops moved houses, and I was sad. I came to the hospital soon. It’s been a while since 2008. I miss this place when I leave. "

(P9, position 115) "For example, when you are out of town and visit a park, you may think of this park or make some comparisons with the park you are in. This situation does happen. If you leave the city, you will still think of the park you often visit. You probably won’t think of this park deliberately. It’s just that I can only think of the park outside when I think of home. For example, if I am in a certain city now, when I walk in the park in that city, I may think of our park. "

Personal Identity

(P13, position 400-408) "If it is complicated, there may be more functions and some changes. I think if it is all regular, it will look very boring sometimes. "

(P23, position 403-404) "You want to retire and go out to learn about society. If you don't work at home, you are out of touch with society, and you are not very clear about social development. I think it is very comfortable and novel outside. You are still in the process of learning in the outside world. If you stay at home, you will not learn. "

(P8, position 62) "People are doing square dancing in the square. There are too many people doing square dancing. I don’t like it because I think it’s a bit noisy. I prefer quieter places. "

(P16, location 25-27)"It's just quiet and unattractive. We just want to bring our kids here and spend our time here, that's all. You can see that there are not many people in this park. There aren't many elderly people who don't like to come here. Some people come here to exercise in the morning, practice swordplay, etc. I don't feel it's very comfortable. "

(P4, position 102-107) " I feel like I need to be younger, because we are 67 now, which means that as I walk along, I feel like I am not yet 61. Yes, you think we should protect this place at all costs. Definitely. "

(P9, position 123) " I just came here to walk around and have a look around. I may not have met many new friends yet. This is something that can only be encountered by chance. It may be due to my personality. "

(P3, position 45-46) "Friends don’t play together. When I ask him out, he says he’ll consider it. When he asks me out, I say I have something to do. We’re all acquaintances. We don’t want to get together anymore. When we were young, we usually got together less and less often. "

(P4, position 86-87) "I didn’t make any new friends in this park. At night, I walked around with my head down, always alone. "

(P7, position 180) " Without any more contact with the outside world, I only have some friends at work. You can imagine that I stay at home all day and have no time to socialise with those people. "

(P8, position 40-41) "I didn’t make any new friends in the park. Very few, almost none. "

(P9, position 60) " I have poor social skills and usually only run into old friends, but I can’t make new friends. "

(P17, position 27) "There are parks everywhere, so there is no need to improve them. They are mainly grain fields. You have not experienced the food panic during that era. If I believe it, it may happen again in the next few years. "

(P22, location 310) "I feel that there are residential areas around the city, so why waste the space? It would be better to give some space to a family to grow some vegetables. "

(P22, position 306) "I still miss home when I first came here. We still don’t want to grow anything at home. We don’t rely on buying and selling. We have our own well water and natural gas. Now our safety is the same as in the city. We all think that the home is almost the same as the outside world. Now we just want to grow crops at home. I think for an ordinary farmer like me, these parks are a waste of space. You think farmers cherish their land, but when I come to these big cities, I see parks like these. One day, we went to where my wife works. They took me to these parks, and I saw that I had dozens of acres of land, but didn’t plant any trees. They just gave up and let the grass grow. I feel that these places in big cities are a waste. "

Preferred spatial features

(P22, location 202) "It's all high-rise buildings. You have to go out to the park to see the green. We don't buy rice or oil at home. We don't buy rapeseed that we grow ourselves. We squeeze oil. We don't buy rice or flour at home. In the countryside, we can raise a pig and kill it during the Spring Festival. We can eat for a year or half a year. We come here. We have to buy side dishes and seasonings every day. You don't have to buy these seasonings in the whole countryside. We grow them ourselves in every household. For example, we have two or three kinds of onions and garlic. Now, if you don't have onions at home in the morning, you have to buy some onions. If you don't have garlic at home, you have to buy some garlic. I think the countryside is still pretty good. "

(P22, position 198) "What can we do with irrigation? We all ride bicycles at home, so here we don’t want to ride even if we have a car. We all walk to exercise our bodies. There are ways to work at home, so we can exercise our bodies. So, do you like to go to the park here and walk around? At home, we have to get up and do something every day. We have to do some side jobs at home, such as growing crops. "

(P22, position 200) " I always pick the spot under that big plane tree; the breeze there feels softer, and I can sit longer"

(P19, position 411) " I like that kind of ecological place. "

(P15, position 18) "I like forests. Forests are good for your mind. "

(P19, location 70) "I like quiet places with nice flowers and plants. We all like places with nice scenery. "

(P22, position 270) "Of course, I miss it, it's like there's some green plant together, it's like the tree is walking here, you still think it's a pity that the tree died, yes, I still cherish it, I still cherish it. "

(P10, location 25) "There is an age range for shade. People over 50 generally prefer places with rich vegetation, while young people prefer squares or lawns. "

(P13, position 386-390) "I like the unique ones. I hope that each place is different and has its own characteristics. The kind that makes people very unexpected. More exploratory and mysterious. "

(P9, position 129) "Like a forest, like when we go to Sanya. When we go to Xishuangbanna, we go to the park, which is like a forest cabin, giving people a very comfortable feeling. This place just feels close, that's all. "

"An ideal park, I think, is first of all, from the perspective of the environment, it must be clean and sanitary. It should have, as I said, I prefer wetland parks. If it is that kind, I like the ones that are bigger and better designed, such as the artificial lake, which is bigger, and the greenery, including some of its facilities, um, more, preferably more hillsides, including some islands in the middle of the lake, and the natural environment, that is, it is closer to the real natural scenery, the real natural environment, closer to the natural scenery, I think that is what I like. "

(P9, position 67) "It is easier to think, usually in a quiet place, sometimes I can chat with friends, or think about some problems by myself. It is quieter, and I like water, so I prefer places with water. I always feel that there are mountains, water and trees, which is the feeling of nature. Since it is a park, it has the feeling of nature. "

(P3, position 96-97) "Mainly because of the good air and quietness. We are annoyed by noise and cannot adapt to it. "

(P9, position 75) "The path should be neatly planned, because this is an urban park after all, not a wild adventure, right? Or a different tourist attraction. If it is a tourist attraction, such as a large tourist attraction, and if the planning is too detailed, it will not be good. I think parks should have a plan. Urban parks should not be too messy. "

(P8, position 81-86) "I still don’t like the ones that are too mysterious. I don’t think they are very comfortable. I think there are no rules, it's just too deep, I just don't like it, maybe because I'm older, I like the brighter ones, the ones with the sun shining brighter. "

(P4, position 41-42) "I like the natural winding and neat ones, it's boring, it's artificially arranged like a river walking, you see there are shade trees here, you see there are sycamore trees, and small branches are quite good, there should be a sense of hierarchy, the actual plants are diverse, there are shrubs, trees, flowers, tea, so it feels like a real garden. "

(P4, position 37) " No, you follow the river. How can you get lost?

It is more primitive, and I can find the original feeling. Road signs are not needed. I walk every day. The beauty of nature. Too much human intervention will make it tasteless. "

(P23, position 89) " I think some larger parks have very clear road signs, so I think you may not get lost. "

(P8, position 78-79) Generally speaking, we prefer clearer ones, with clearer road signs. Otherwise, what if we get lost when we are old? "

(P8, position 89) "If bushes are too high, I'd rather sit in the open where I can see people coming"

(P2, position 38-44) " I don't like the kind of circuitous and hidden ones; I like the refreshing ones. I don't think there is a need for too many changes. I think the kind that is clear at a glance is better, that is, the road is easy to identify the direction. If it is too complicated, it will not work. I don't think it is so good. "

(P11, location 33-41) "What kind of space do you think is most likely to restore physical and mental health? It is by the water, especially by the windmill. Or there is a large dam next to the pier, which is very open. It is very wide, you can sit there, and the air is good and the view is good. "

(P4, position 185-187) " Yes, it flows with the water. If you build a canal like a rural irrigation canal at the gate, the sound of the water flowing can also enter nature, which is better. The natural environment circulates it, or it is mechanically raised a little to keep it so that it is more vibrant. When it stays, it can be like a child running there to catch small fish and shrimp. "

(P16, location 25-27) " The scenery here is quite diverse. For example, the park has trees, lawns, and ornamental plants. There are at least hundreds of species of plants. One is that you can recognise plants and see various flowers and plants. Although it has different seasons, for example, in this season, it has bougainvillaea and bougainvillaea. In winter, there are also magnolias, crabapple flowers, and magnolias, so there are many varieties of flowers here. There is also a relatively quiet bamboo forest, which is the bamboo forest of Tazishan Park. "

1.4 Data Coding Process Example

The coding process employed a combination of top-down deductive and bottom-up inductive approaches, integrating predefined theoretical concepts while remaining open to emergent patterns in the data. The first stage is open coding. Using line-by-line and word-by-word analysis of the interview transcripts, three frequently mentioned concepts were identified and coded as free nodes in the initial phase of thematic analysis, as shown in Figures 1 to 3.

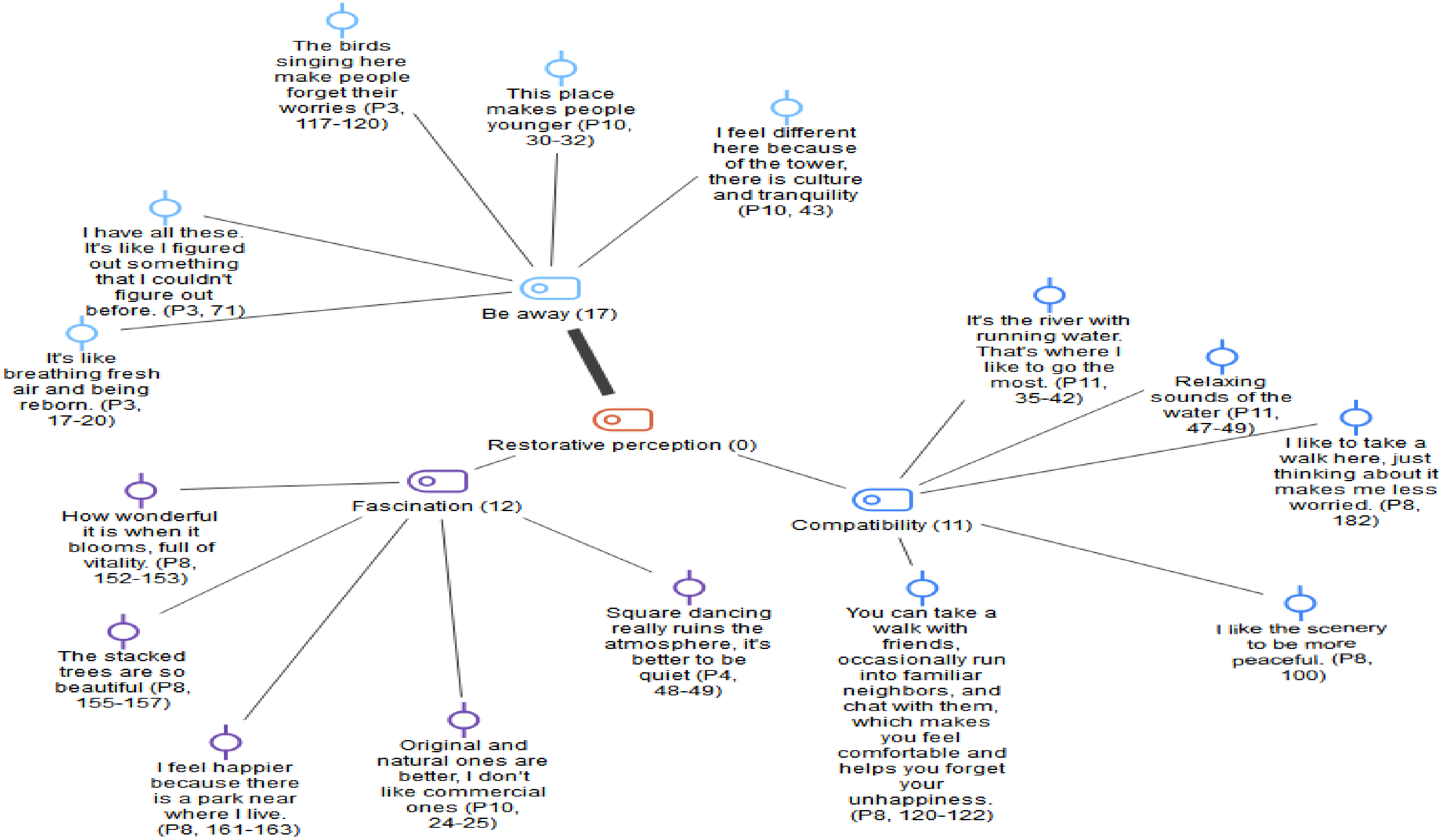

Supplementary Figure S1. Coding Part 1.

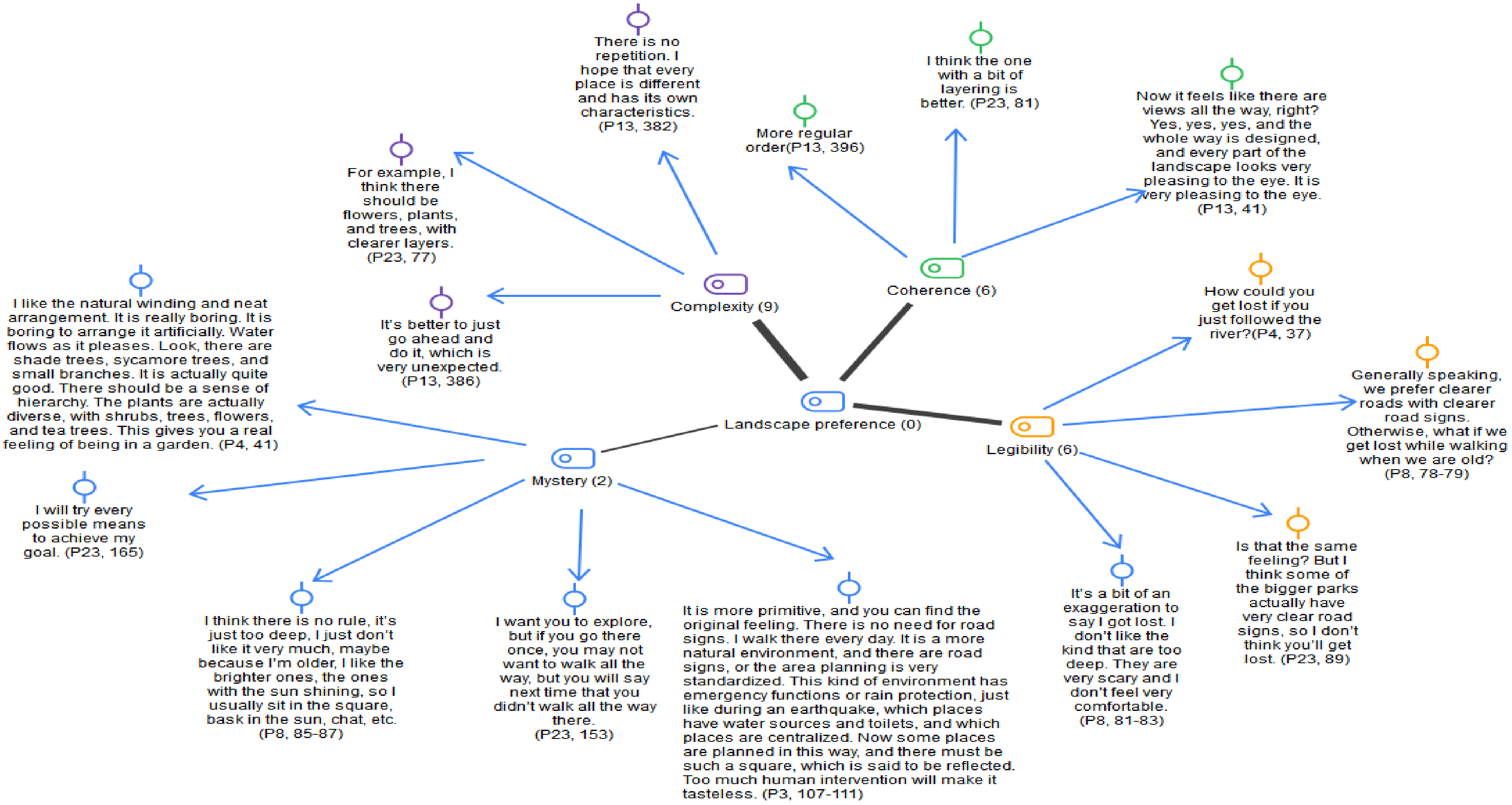

Supplementary Figure S2. Coding Part 2.

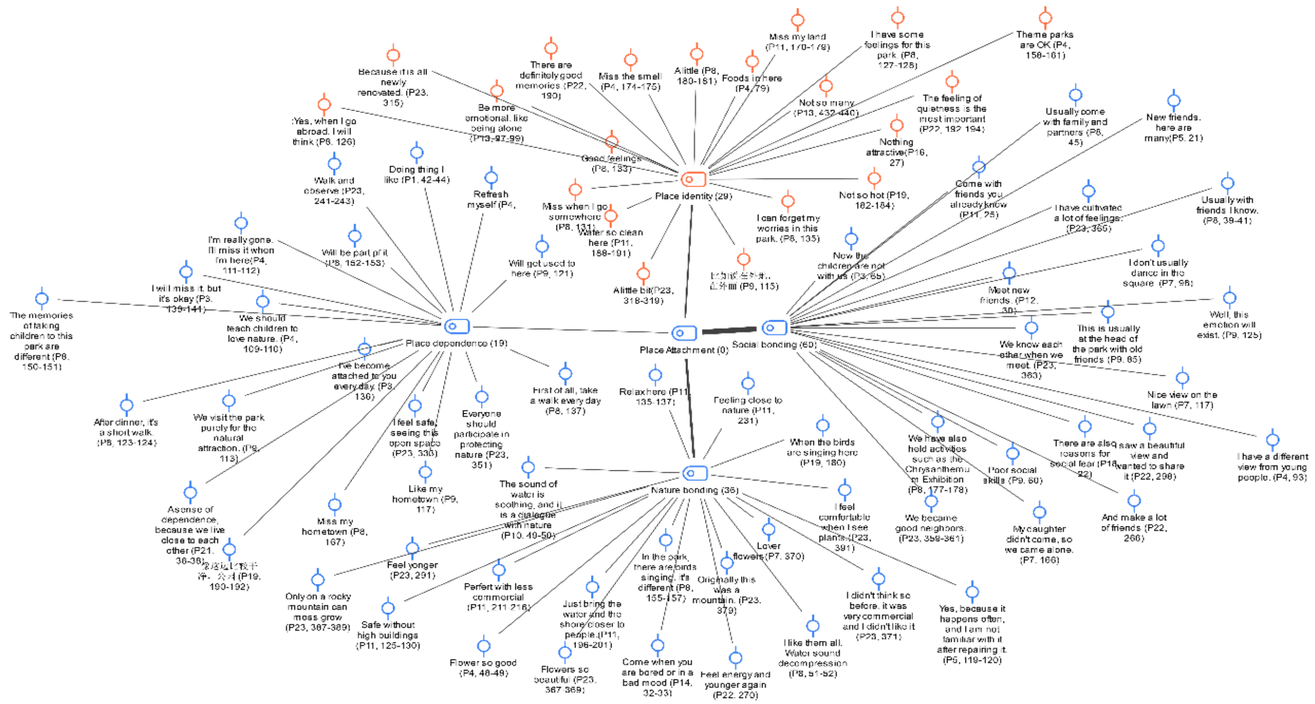

Supplementary Figure S3. Coding Part 3.

The second stage, Axial coding, involves examining associations among the existing free coding nodes. The aim is to group similar or related concepts and condense them into fewer, higher-level categories. Through iterative comparisons and analyses, the most prominent categories are identified and designated as the main factors influencing the study's themes. Axial coding is based on the open coding seen in Appendix Table 2.

The third stage is Selective coding, which synthesises the findings from the previous stages by identifying core categories to construct a more systematic and cohesive conceptual framework.

Building on the deductive approach employed in the first stage and the inductive approach utilised in the second stage, this phase consolidates findings into a coherent summary. The core categories are selected to form a more systematic concept. Three main factors influencing perceptual recovery were identified: individual needs (differences in activities and spatial usage), subjective perception (human-land attachment). Landscape quality (variations in user preferences).

According to the third stage, the model was established. After the three-stage coding of the landscape, the logical relationship between nodes and sub-nodes was formed.

For instance, in the coding tree, concepts such as 'Layered' or 'Colourful,' derived from open coding (S1), were grouped into the broader category 'Rich and Orderly' (S2). This category ultimately contributed to a core theme in the final analysis (Phase 3), characterised as 'Rich, Mysterious, and Orderly.' It evokes a desire to explore while fostering a sense of security, control, and accomplishment.

For example, in the coding tree, "Humanities and history" or "Musical instruments" is an axis coding generated by open coding (S1), which then forms the broader category. The history on display and the sounds and smells provide a welcome break from the daily grind" (S2).

This category corresponds to one of the main themes of the final analysis (Phase 3), highlighting the richness in both tangible and intangible elements of the park environment, evoking a strong sense of 'identity' among participants.

Once the final coding scheme was established, the initially coded interviews were systematically reviewed and documented to ensure alignment with the identified themes. This meant that the analytical material was organised around three main themes, matching the influences of recovery.

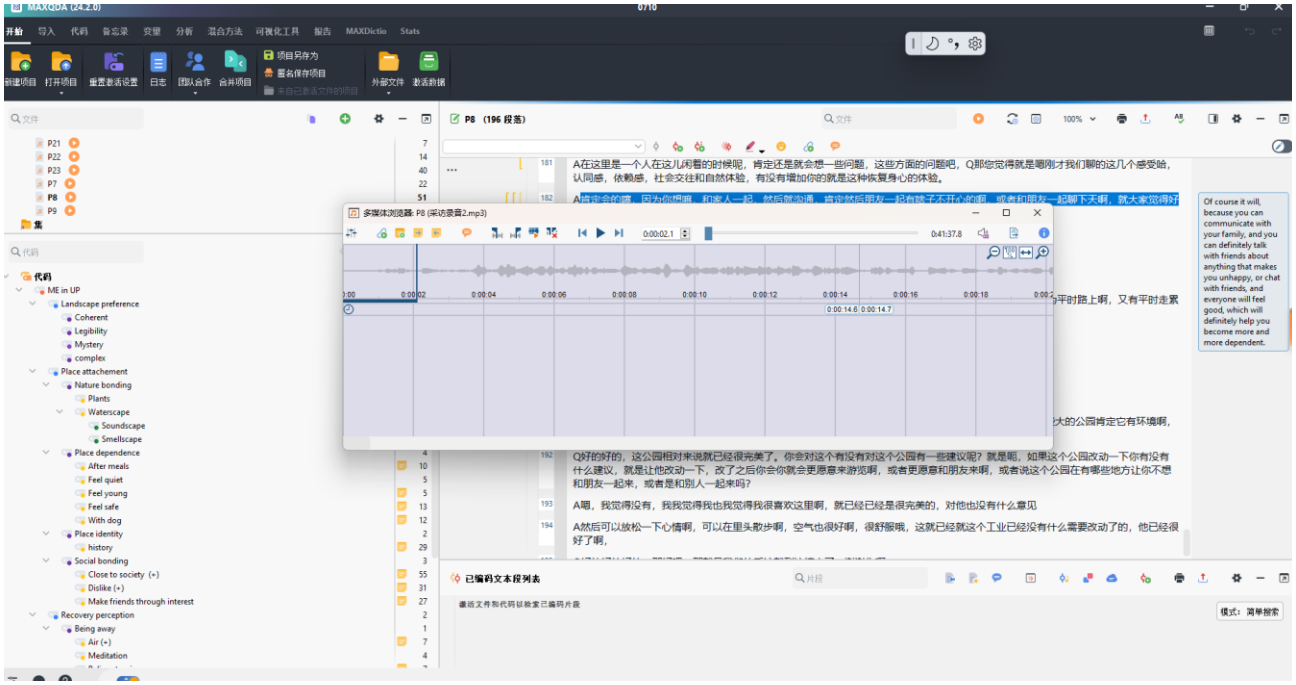

Supplementary Figure S4. Screenshot of MAXQDA interface, showing the coding process (associated audio recordings are available upon request).
